# Association Between Dietary Vitamin K Intake With Cancer Cachexia and Mortality: NHANES 1999–2018

**DOI:** 10.1002/fsn3.70917

**Published:** 2025-10-28

**Authors:** Guoming Chen, Siran Lai, Geer Lin, Tianyi Li, Xiangjun Qi, Cheng Zhang, Guang Chen, Guoyi Tang, Ning Wang, Yibin Feng

**Affiliations:** ^1^ School of Chinese Medicine, Li Ka Shing Faculty of Medicine The University of Hong Kong Hong Kong SAR China; ^2^ The First Clinical Medical School of Guangzhou University of Chinese Medicine Guangzhou China; ^3^ Foshan Clinical Medical College, Guangzhou University of Chinese Medicine Guangzhou China; ^4^ School of Public Health and Management, Guangzhou University of Chinese Medicine Guangzhou China

**Keywords:** cancer cachexia, dietary risk factors, mortality, NHANES, vitamin K

## Abstract

Research on the association between the development of cancer cachexia and dietary nutrient intake remains insufficient. We discussed the screening of dietary intake risks for cancer cachexia and further investigated the impact of vitamin K on the prognosis of cancer cachexia survivors. We ultimately analyzed 3489 NHANES participants across 10 cycles. Multivariate logistic regression analysis and restricted cubic spline (RCS) methods were used to explore the relationship between the intake of most dietary nutrients and the incidence of cancer cachexia. Further, multivariate Cox regression analysis, Kaplan–Meier survival analysis, and RCS analysis were employed to examine the association between vitamin K intake and mortality among cancer cachexia survivors. The multivariate logistic regression model revealed that vitamin K, dietary fiber, β‐carotene, food folate, and magnesium might increase the risk of cancer cachexia. In contrast, energy, α‐carotene, retinol, lycopene, iron, and copper might reduce the risk of cancer cachexia. Further RCS analysis showed an inverse U‐shaped relationship between vitamin K intake and the incidence of cancer cachexia. Multivariate Cox regression analysis indicated that the high vitamin K intake group could reduce the risk of all‐cause mortality (HR = 0.71; 95% CI, 0.60–0.84; *p* < 0.0001) and cardiac mortality (HR = 0.72; 95% CI, 0.54–0.84; *p* = 0.03) among cancer cachexia survivors. The intake of various dietary nutrients is associated with the incidence of cancer cachexia. In particular, there is an inverse U‐shaped relationship between vitamin K intake and the incidence of cancer cachexia, and high vitamin K intake may reduce the risk of all‐cause and cardiac mortality in cancer cachexia survivors.

## Graphical abstract

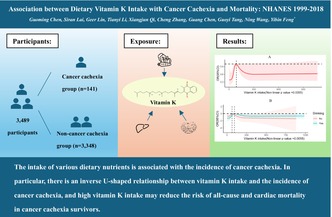

**Abbreviations**
95% CI: 95% confidence interval
BMI: Body mass index
HR: Hazard ratio
ICD‐10: International Classification of Diseases, 10th10th Revision
MEC: Mobile Examination Center
NHANES: National Health and Nutrition Examination Survey
OR: Odds ratio
PIR: Poverty income ratio
RCS: Restricted cubic spline
SE: Standard errors

## 1 Introduction

“Cachexia” comes from kakos, meaning “bad” and hexis, for “habit.” Cancer Cachexia is a nutritional deficiency often found in individuals battling long‐term diseases such as cancer, heart failure, renal failure, and autoimmune disorders (Nishikawa et al. 2021). Cancer cachexia, characterized as a complex disorder, is identified by continuous depletion of muscle tissue (either solely or alongside adipose tissue loss). This condition is not entirely remediable through standard nutritional interventions and results in escalating functional decline. Advanced cancer patients frequently experience cancer cachexia. For cancer patients, cancer cachexia is identified by a weight loss of greater than 5%, and in those with a body mass index (BMI) below 20 kg/m^2^ or sarcopenia, even a 2% loss suffices for diagnosis (Fearon et al. 2011). Cachexia in cancer is caused by a combination of factors: diminished nutritional intake paired with metabolic disturbances such as higher energy expenditure, muscle wasting, and inflammatory responses (Baracos et al. 2018). Cancer cachexia has affected 50%–80% of patients with cancer and is responsible for approximately 20% of all cancer‐related fatalities (Argilés et al. 2023). As high as its incidence and death rates are, no cure exists to counteract its effects (Kim et al. 2021). Therefore, identifying cancer cachexia risk factors is crucial for timely intervention and delay of disease onset.

Investigations into the causes of cancer cachexia have recognized several influences, including reduced appetite, increased energy expenditure, and metabolic disturbances, where eating patterns and nutrient assimilation play a significant role (Argilés et al. 2014; Fearon et al. 2011). The link between diet and cancer cachexia is thus becoming a subject of greater scrutiny. The link between cancer cachexia and diet has garnered significant attention from scholars worldwide. However, current studies have predominantly focused on dietary treatment plans for patients with cancer cachexia (Kim et al. 2021). Little research has addressed dietary factors that precipitate cancer cachexia. Limited research exists on how vitamin K consumption affects mortality among patients with cancer cachexia. To combat cancer cachexia, investigation of its dietary determinants and the influence of vitamin K on patient mortality is imperative.

Originally discovered for its hemorrhage‐prevention properties nearly a century ago, vitamin K is increasingly recognized for its broader impact on numerous physiological functions. However, its role in cancer cachexia remains uncertain. A study showed that vitamin K has a potential anticancer effect in prostate cancer (Ivanova et al. 2018). Data from the Heidelberg study (Nimptsch et al. 2008) showed an inverse association between cancer incidence and vitamin K2 intake in men, and an inverse association was shown between dietary vitamin K2 intake and mortality from lung and prostate cancer. By contrast, the total vitamin K intake was associated with an increased risk of breast cancer and death from breast cancer, which was shown in a prospective cohort study (Wang et al. 2021). Another analysis also revealed a positive association between vitamin K intake and cancer incidence (Qin et al. 2025). Although previous studies have investigated the association between vitamin K and cancer, research on vitamin K and cancer cachexia remains limited and insufficiently comprehensive. Existing analyses seldom isolate vitamin K intake when investigating its association with cancer cachexia. Therefore, we examined the association between vitamin K intake and both incidence and mortality of cancer cachexia.

The focus of this manuscript is to conduct a detailed analysis of dietary risks in the context of cancer cachexia and to investigate the association between vitamin K intake and both incidence and mortality of cancer cachexia. By elucidating the interplay between dietary elements and the outcomes of cancer cachexia, our goal is to provide insightful perspectives that may guide the development of tailored nutritional interventions to enhance the management of this debilitating syndrome. This research area is relatively novel and essential. The parameters for assessment in this research include the evaluation of disease progression, patient survival, and the quality of life among individuals with cancer cachexia (Bauer et al. 2002; Bruera and Hui 2010). Our objectives are to pinpoint the dietary factors that increase the risk of cancer cachexia and to study the correlation between vitamin K intake and death rates in affected patients.

## 2 Materials and Methods

The National Health and Nutrition Examination Survey (NHANES) is an extensive research program that evaluates the health and dietary habits of both adults and children across the United States. NHANES has been a pivotal resource since the 1960s, evolving into an ongoing program that has systematically collected data from an average of 5000 participants each year. This data encompasses a wide array of information, including demographic, dietary, clinical, laboratory, survey data, and select restricted access datasets.

### 2.1 Study Population

**FIGURE 1:**
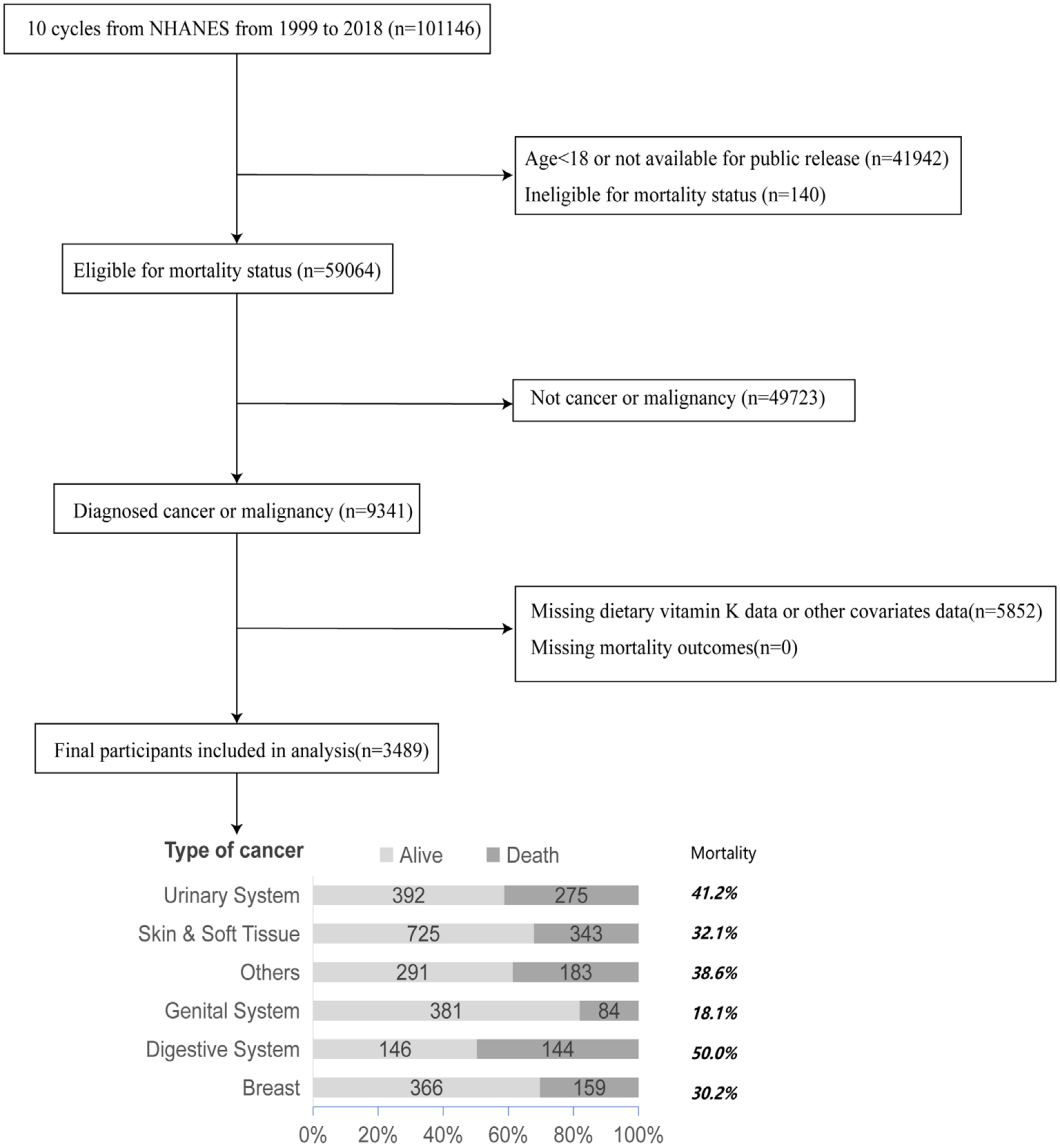
Flowchart of participants selection.

The data we utilized originated from NHANES, a research initiative specifically crafted to evaluate the health and nutritional standing of individuals within the United States. This study included data from 10 NHANES cycles (1999–2018) embodying a total of 101,146 participants. Initially, the dataset was pruned to exclude records of individuals under the age of 18, as their data could not be publicly disclosed (*n* = 41,942), as well as those with invalid death data (*n* = 140). Subsequently, participants without cancer were excluded from the study (*n* = 49,723). Further refinement of the cohort involved the removal of cases with missing values for vitamin K and covariates, which included age, ethnicity, gender, education level, BMI, poverty‐to‐income ratio (PIR), marital status, diabetes status, hypertension status, smoking status, and drinking status (*n* = 5852). Ultimately, a total of 3489 adult participants meeting the selection criteria were included in the analytical assessment. The systematic process of subject selection is depicted in Figure 1.

### 2.2 Outcomes and Exposure Assessment

The principal outcome under investigation was the prevalence of cancer cachexia among the study participants. This was ascertained through self‐reported health status data obtained during personal interviews as part of the NHANES Medical Condition section (prefix MCQ). (1) Participants who affirmed to the query “Has a physician or another healthcare provider ever informed you that you have been diagnosed with cancer, including any form of cancer cachexia?” (variable mcq220); (2) Participants who responded to the inquiry “What type of cancer do you have?” (variable mcq230a) were identified as having cancer. In accordance with the prevailing diagnostic criteria for cancer cachexia, which is defined by the presence of cancer and a BMI below 20. Participants with cancer and a BMI under 20 were classified as a cancer cachexia group (Anker et al. 2019; Fearon et al. 2011; Lee et al. 2023), while those with cancer and a BMI of 20 or higher were classified as a noncancer cachexia group for the subsequent diet‐related analyses (Arends et al. 2021). The NHANES database provides a detailed classification of cancer into up to 45 distinct types. We systematically summarized and categorized the types of cancer for the overall population with cancer, cancer cachexia, and noncancer cachexia in tabular format. Cancers were systematically grouped into six types based on the responses to variable mcq230a, ensuring a relatively balanced distribution across malignant skin and soft tissue tumors, as well as cancers of the urinary system, reproductive system, digestive system, and breast. Participants without any of the aforementioned cancers were categorized separately (Table S1). Table S2 provides a detailed breakdown of the definitions and numbers of deaths, and we performed a comprehensive summary analysis of the causes and numbers of deaths for the overall population with cancer, cancer cachexia, and noncancer cachexia.

The intake of vitamin K was the primary exposure variable in this study. The dietary interview section was designed to elicit detailed information regarding the dietary intake of NHANES participants. The dietary intake data served to calculate the variety and quantities of food and beverages consumed in the 24 h preceding the interview (from midnight to midnight), along with the intake of nutrients, energy, and additional elements found in these sources. For each NHANES participant, the total daily intake of energy and nutrients from food and beverages, as well as the quantity of food that is typically consumed, significantly more or significantly less compared to the normal level, was all documented in the total nutrient intake file. Every participant in the NHANES was qualified to take part in two 24‐h dietary recall interviews. The primary dietary recall interview was held on‐site at the Mobile Examination Center (MEC), with the subsequent interview conducted via telephone 3–10 days later. The intake levels of vitamin K were then segmented into quartile categories. In this study, we focused on the vitamin K (mcg) intake from the overall nutrient consumption on the initial day, which was collected in person by the MEC during the initial 24‐h recall interview.

### 2.3 Mortality Outcomes

National Death Index connecting with mortality data was by NHANES up to December 31, 2019. According to the International Classification of Diseases, 10th Revision (ICD‐10), cancer mortality was classified as deaths caused by malignant tumors (ICD‐10:C00‐C97). The study examined both cause‐specific mortality and all‐cause mortality, encompassing heart disease (ICD‐10: I00‐I09, I11, I13, I20‐I51) and malignant tumors (ICD‐10: C00‐C97) (Lee et al. 2023). Calculating survival time began with the baseline for NHANES data collection as the starting point. The duration of follow‐up was determined by the period commencing from the interview date and extending to either the date of demise for those who passed away, or up until December 31, 2019, for those participants who did not encounter the event in question.

### 2.4 Covariates Assessment

We conducted a thorough screening of ten potential confounding factors associated with cancer cachexia, which included age, race, gender, smoking status, poverty income ratio, education level, marital status, diabetes status, hypertension status, and drinking status, all of which were recognized as risk factors. The age range of the participants was from 20 to 85 years old. BMI was calculated by dividing an individual's weight in kilograms by the square of their height in meters with a range of 13.36–32.6. Race was classified into Mexican Americans, other Hispanics, non‐Hispanic blacks, non‐Hispanic whites, and other races. Gender was dichotomized into male and female. Education level was categorized into three groups: less than high school, high school, and more than high school. PIR were classified into three parts: PIR ≤ 1.0, 1.0 < PIR ≤ 3.0, and PIR > 3.0. Self‐reported chronic diseases, including hypertension and diabetes (diagnosis can be made if any of the following conditions are met: being diagnosed by a doctor or health professional; hemoglobin A1c ≥ 6.5%; fasting blood glucose ≥ 7.0 mmol/L; casual blood glucose ≥ 11.1 mmol/L; 2‐h glucose (oral glucose tolerance test) ≥ 11.1 mmol/L; using anti‐diabetic medication. Individuals with impaired glucose tolerance and impaired fasting glucose, including who identified themselves as being in “borderline” conditions, were not considered to have diabetes), and hypertension (was defined as follows: (1) an average systolic pressure of 130 mmHg or higher and/or an average diastolic pressure of 80 mmHg or higher; (2) self‐reported hypertension history; (3) using medication for anti‐hypertension). Height, weight, and drinking status were recorded at the Mobile Examination Center. Drinking status was defined as the consumption of at least 12 drinks per year. Individuals who either have never smoked or have smoked less than 100 cigarettes throughout their entire life were categorized as nonsmokers.

### 2.5 Statistical Analysis

Statistical analyses were entirely performed using R 4.2.0, employing the complex survey weights recommended by the Centers for Disease Control and Prevention (CDC) for the initial day of dietary samples. We integrated the sample weights across ten consecutive cycles following the methodologies endorsed by NHANES. In the table outlining baseline characteristics, continuous variables were shown as survey‐weighted means (standard errors), and categorical variables were indicated by sample sizes (survey‐weighted percentages).

To thoroughly investigate the correlation between cancer cachexia and vitamin K, we performed both association and survival analyses. For association analysis between the incidence of cachexia and vitamin K, we used one survey‐weighted generalized linear model with unadjusted covariates and three multivariate models adjusting for different covariates. The base model was unadjusted. Model 1 was adjusted for only age. Model 2 included adjustments for age, education level, gender, race, PIR, and marital status. Model 3 was further modified for hypertension status, smoking status, drinking status, and diabetes status. We employed restricted cubic spline (RCS) curves to inspect the dose–response association between the prevalence of cancer cachexia and vitamin K intake. Analyses utilizing multivariate logistic regression and subgroup methods were conducted to explore the association between vitamin K consumption, evaluated as a continuous variable as well as a categorical one (divided into quartiles), and the incidence of cancer cachexia. Subgroup analyses were stratified by age, marital status, gender, race, status, education level, hypertension status, poverty income ratio, status, diabetes status, smoking status, and drinking status to assess the consistency of the association between cancer cachexia prevalence and intake of vitamin K across different subgroups.

For examining the impact of vitamin K on cancer cachexia in terms of survival analysis, we implemented the Kaplan–Meier technique to evaluate survival and the Survey‐weighted Cox models to measure the strength of the association between vitamin K intake and all‐cause mortality, heart disease mortality, and cancer mortality. To ascertain and illustrate the dose–response link between vitamin K intake and the risk of death, we made use of models based on restricted cubic splines, with RCS analysis conducted for cancer mortality, heart disease mortality, and all‐cause mortality. To assess whether a non‐linear association existed, we compared models incorporating cubic spline expressions to those without such terms using the likelihood ratio test. Subgroup analyses were additionally performed to examine the correlation between vitamin K consumption and the risk of death among different demographic or clinical subgroups, stratified by age, gender, marital status, race, poverty income ratio, education level, hypertension status, smoking status, diabetes status, and drinking status. Multiplicative interaction terms were included in the models to evaluate potential interaction effects. The study regards all statistical analyses as statistically significant with a *p*‐value threshold below 0.05.

## 3 Results

### 3.1 Characteristics of Participants

A total of 101,146 subjects from ten cycles (1999–2018) of the NHANES database were enrolled in our study. After screening, 3489 participants were ultimately encompassed in analyses. Based on intake of daily dietary vitamin K, a weighted baseline table for the population was created with quartile classification (Table 1). The finally included participants can be divided into the non‐cancer cachexia group (*n* = 3348) and the cancer cachexia group (*n* = 141). We conducted a comprehensive analysis of factors that may be associated with the occurrence of cancer cachexia, including routine dietary intake (energy, protein, carbohydrates, sugar, dietary fiber, fat, and cholesterol), vitamin family (retinol, vitamin A, alpha‐carotene, beta‐cryptoxanthin, beta‐carotene, zeaxanthin, lycopene, vitamin B1, niacin, vitamin B2, vitamin B6, folic acid, total folate, food folate, vitamin B12, vitamin C, vitamin K, vitamin E), microelement (phosphorus, calcium, zinc, magnesium, iron, copper, selenium, sodium, and potassium), age, race, gender, PIR, education level, marital status, diabetes status, smoking status, hypertension status, and drinking status. The average age (SE) of the participants was 62.22 (0.38) years, with an average dietary vitamin K intake (SE) of 122.25 (10.82) mcg/day. The majority of the population, 57.02%, were female, 87.87% were non‐Hispanic whites, 74.66% had a high education level, 57.50% had a PIR greater than 3.0, 66.54% were married or cohabitating, 69.86% were smokers, 58.35% were drinkers, 21.04% had diabetes, and 58.35% had hypertension. We also summarized the basic characteristics of the cancer cachexia population, which were generally similar to the overall cancer population. The mean age (SE) of the cancer cachexia population was 62.37 (1.58) years, with an average dietary vitamin K intake (SE) of 130.09 (22.32) mcg/day (Table S2). The weighted incidence rate of cancer cachexia in this investigation was 4.15%, with a higher prevalence of cancer cachexia in the group of high vitamin K intake compared to the group of low vitamin K intake (Q1, 2.64%; Q2, 4.14%; Q3, 4.76%; Q4, 4.85%), with their basic characteristics shown in Table 1.

**TABLE 1:** Baseline characteristics among cancer patients according to vitamin K intake.

Variables	Vitamin K intake					*p*
	Overall	Q1	Q2	Q3	Q4	
*N*	3489	872	872	872	872	
Age, years	62.22 (0.38)	59.61 (0.69)	62.76 (0.74)	63.98 (0.61)	62.34 (0.60)	< 0.0001
Sex, *n* (%)						< 0.001
Female	1817 (57.02)	522 (65.12)	449 (56.71)	407 (50.72)	439 (56.24)	
Male	1672 (42.98)	353 (34.88)	423 (43.29)	463 (49.28)	433 (43.76)	
Race, *n* (%)						0.31
Non‐Hispanic white	2506 (87.87)	612 (86.77)	629 (87.62)	644 (89.07)	621 (87.91)	
Non‐Hispanic black	475 (5.14)	130 (5.77)	101 (4.62)	106 (4.80)	138 (5.37)	
Mexican American	206 (1.93)	63 (2.03)	67 (2.97)	42 (1.54)	34 (1.33)	
Other race	302 (5.05)	70 (5.42)	75 (4.80)	78 (4.58)	79 (5.39)	
Education level, *n* (%)						< 0.0001
Less than high school	314 (4.62)	116 (7.28)	85 (5.36)	64 (3.89)	49 (2.46)	
High school	910 (20.72)	281 (27.65)	231 (20.99)	240 (22.72)	158 (12.98)	
More than high school	2265 (74.66)	478 (65.07)	556 (73.66)	566 (73.40)	665 (84.56)	
Poverty income ratio, *n* (%)						< 0.0001
<=1.0	469 (9.30)	180 (15.23)	108 (10.29)	89 (6.32)	92 (6.24)	
1.0–3.0	1494 (33.20)	404 (38.04)	409 (37.29)	366 (33.12)	315 (25.81)	
> 3.0	1526 (57.50)	291 (46.73)	355 (52.42)	415 (60.56)	465 (67.95)	
Marriage, *n* (%)						0.06
Divorced/separated/widowed	1146 (28.44)	310 (31.87)	281 (29.23)	267 (27.52)	288 (25.76)	
Married/living with partner	2148 (66.54)	504 (61.38)	548 (65.88)	550 (67.36)	546 (70.65)	
Never married	195 (5.01)	61 (6.75)	43 (4.88)	53 (5.12)	38 (3.59)	
Energy intake (kcals/day)	1955.61 (21.95)	1478.08 (24.94)	1927.20 (29.77)	2140.23 (42.85)	2209.51 (41.43)	< 0.0001
Protein intake (g/day)	74.93 (0.86)	56.14 (1.45)	73.43 (1.60)	81.75 (1.58)	85.66 (1.66)	< 0.0001
Carbohydrate intake (g/day)	232.84 (2.97)	186.32 (3.83)	234.30 (4.50)	252.36 (5.11)	252.65 (5.40)	< 0.0001
Sugar intake (g/day)	106.48 (1.95)	92.38 (2.95)	104.91 (3.28)	111.78 (2.99)	114.72 (3.70)	< 0.0001
Fiber intake (g/day)	16.47 (0.27)	10.01 (0.32)	15.35 (0.44)	18.13 (0.51)	21.27 (0.48)	< 0.0001
Tfat intake (g/day)	77.30 (1.04)	51.55 (1.15)	74.79 (1.57)	86.21 (2.16)	92.74 (2.06)	< 0.0001
Sfat intake (g/day)	25.35 (0.40)	18.71 (0.53)	25.33 (0.63)	28.10 (0.85)	28.38 (0.79)	< 0.0001
Mfat intake (g/day)	27.58 (0.37)	18.60 (0.45)	27.16 (0.61)	30.87 (0.84)	32.40 (0.79)	< 0.0001
Pfat intake (g/day)	17.45 (0.28)	9.26 (0.23)	15.54 (0.34)	19.57 (0.50)	23.93 (0.62)	< 0.0001
Cholesterol intake (mg/day)	265.63 (5.30)	204.62 (7.47)	265.42 (10.57)	288.48 (10.49)	295.84 (9.90)	< 0.0001
Vitamin A intake (mcg/day)	677.34 (18.90)	442.26 (31.20)	570.22 (20.49)	695.33 (20.52)	946.17 (48.36)	< 0.0001
Retinol intake (mcg/day)	443.88 (10.92)	358.12 (26.61)	440.69 (19.51)	486.65 (16.13)	479.24 (17.35)	0.001
Alpha‐carotene intake (mcg/day)	471.68 (44.68)	120.42 (18.49)	313.02 (37.47)	458.79 (39.92)	907.81 (149.00)	< 0.0001
Beta‐carotene intake (mcg/day)	2520.13 (151.97)	918.03 (165.68)	1356.47 (89.97)	2222.12 (148.58)	5094.72 (469.85)	< 0.0001
Beta‐cryptoxanthin intake (mcg/day)	97.40 (3.98)	63.79 (6.08)	89.57 (7.29)	112.48 (8.64)	118.33 (8.58)	< 0.0001
Lycopene intake (mcg/day)	4855.42 (209.74)	2660.69 (316.61)	4058.36 (332.17)	6232.87 (467.70)	6110.68 (459.65)	< 0.0001
Zeaxanthin intake (mcg/day)	1663.66 (93.63)	423.06 (22.76)	728.62 (33.17)	1043.39 (29.73)	4034.81 (297.69)	< 0.0001
Vitamin B1 intake (mg/day)	1.55 (0.02)	1.17 (0.03)	1.56 (0.04)	1.66 (0.04)	1.74 (0.04)	< 0.0001
Vitamin B2 intake (mg/day)	2.15 (0.03)	1.75 (0.05)	2.13 (0.05)	2.26 (0.05)	2.39 (0.05)	< 0.0001
Niacin intake (mg/day)	22.64 (0.27)	17.56 (0.53)	22.35 (0.57)	24.75 (0.49)	25.18 (0.54)	< 0.0001
Vitamin B6 intake (mg/day)	1.90 (0.03)	1.42 (0.06)	1.82 (0.05)	2.03 (0.05)	2.24 (0.05)	< 0.0001
Total folate intake (mcg/day)	386.18 (5.68)	278.61 (8.43)	358.06 (9.26)	411.03 (10.04)	476.69 (11.20)	< 0.0001
Folic acid intake (mcg/day)	172.33 (3.49)	146.36 (6.78)	171.16 (5.77)	192.11 (7.85)	177.10 (6.33)	< 0.001
Food folate intake (mcg/day)	213.90 (3.99)	132.27 (3.84)	186.95 (7.46)	219.09 (4.31)	299.54 (8.54)	< 0.0001
Vitamin B12 intake (mg/day)	4.95 (0.10)	4.18 (0.21)	4.95 (0.24)	5.26 (0.18)	5.29 (0.20)	0.001
Vitamin C intake (mg/day)	82.04 (2.20)	45.00 (2.79)	68.50 (3.04)	84.69 (3.35)	121.75 (6.07)	< 0.0001
Vitamin E intake (mg/day)	8.09 (0.14)	4.28 (0.15)	6.92 (0.19)	9.17 (0.29)	11.27 (0.31)	< 0.0001
Calcium intake (mg/day)	891.05 (14.14)	697.67 (23.95)	864.59 (25.86)	949.90 (27.36)	1020.73 (26.18)	< 0.0001
Phosphorus intake (mg/day)	1274.71 (15.97)	968.92 (22.45)	1240.90 (26.47)	1373.10 (30.38)	1468.23 (28.76)	< 0.0001
Magnesium intake (mg/day)	288.26 (3.50)	207.02 (4.55)	271.43 (5.46)	306.70 (6.13)	353.17 (7.61)	< 0.0001
Iron intake (mg/day)	14.55 (0.18)	11.07 (0.27)	14.25 (0.31)	15.84 (0.36)	16.53 (0.38)	< 0.0001
Zinc intake (mg/day)	10.88 (0.15)	8.44 (0.25)	10.72 (0.23)	11.87 (0.30)	12.14 (0.27)	< 0.0001
Copper intake (mg/day)	1.24 (0.02)	0.86 (0.03)	1.19 (0.04)	1.32 (0.03)	1.54 (0.04)	< 0.0001
Sodium intake (mg/day)	3177.31 (36.39)	2281.35 (48.61)	3023.32 (50.80)	3521.44 (75.68)	3740.60 (78.45)	< 0.0001
Potassium intake (mg/day)	2667.95 (32.93)	1877.64 (41.61)	2532.14 (52.68)	2898.71 (53.38)	3230.00 (66.71)	< 0.0001
Selenium intake (mg/day)	101.81 (1.27)	75.48 (1.82)	99.25 (2.36)	112.54 (2.29)	116.14 (2.64)	< 0.0001
Drinking status, *n* (%)						< 0.0001
No	1306 (30.14)	378 (35.50)	347 (32.18)	323 (31.25)	258 (22.97)	
Yes	2183 (69.86)	497 (64.50)	525 (67.82)	547 (68.75)	614 (77.03)	
Diabetes mellitus, *n* (%)						0.73
No	2593 (78.96)	627 (78.91)	641 (77.56)	665 (80.44)	660 (78.85)	
Yes	896 (21.04)	248 (21.09)	231 (22.44)	205 (19.56)	212 (21.15)	
Hypertension, *n* (%)						0.34
No	1249 (41.65)	305 (43.65)	313 (41.65)	306 (38.02)	325 (43.23)	
Yes	2240 (58.35)	570 (56.35)	559 (58.35)	564 (61.98)	547 (56.77)	
Smoking status, *n* (%)						0.05
No	1507 (44.50)	340 (38.61)	398 (45.74)	377 (44.87)	392 (48.01)	
Yes	1982 (55.50)	535 (61.39)	474 (54.26)	493 (55.13)	480 (51.99)	
Vitamin K intake (mcg/day)	122.25 (10.82)	21.09 (0.41)	48.74 (0.35)	86.42 (0.77)	300.08 (37.15)	< 0.0001
Cancer cachexia, *n* (%)						< 0.001
No	3348 (95.85)	846 (97.36)	841 (95.86)	827 (95.24)	834 (95.15)	
Yes	141 (4.15)	29 (2.64)	31 (4.14)	43 (4.76)	38 (4.85)	

*Note:* Continuous variables are shown as weighted means ± standard errors. Categorical variables are shown as unweighted counts (weighted percentages). Q1, ≤ 34.5 mcg/day; Q2, 34.5–62.3 mcg/day; Q3, 62.3–118.1 mcg/day; Q4, ≥ 118.1 mcg/day.

In our investigation, we meticulously categorized the study cohort based on the type of cancer and subsequently analyzed the associated mortality rates. The distribution of cancer types among the participants was as follows: patients with skin or soft tissue cancers numbered 1068 (32.1%), those with urinary system cancers accounted for 667 (41.2%), breast cancer patients totaled 525 (30.2%), reproductive system cancer patients were recorded as 465 (18.1%), and patients with digestive system cancers amounted to 290 (50%). As delineated in Figure 1, the cancer type with the highest mortality rate was digestive system cancer, followed closely by cancers of the urinary system. A detailed breakdown of the cancer classifications and the corresponding patient numbers is presented in Table S1.

### 3.2 Association Between Vitamin K Intake, Other Dietary Intakes, and Cancer Cachexia

The association between vitamin K intake, other dietary intakes, and cancer cachexia was evaluated using survey‐weighted generalized linear models. After survey‐weighted generalized linear models of most dietary intakes, we found that vitamin K, dietary fiber, beta‐carotene, food folate, and magnesium were positively correlated with cancer cachexia, suggesting that these dietary factors may increase the risk of cancer cachexia (Table 2). Conversely, energy, alpha‐carotene, retinol, lycopene, iron, and copper were negatively correlated with cancer cachexia, indicating that these dietary factors may reduce the risk of cancer cachexia (Table 2). Meanwhile, we observed little association between routine dietary intake (protein, carbohydrates, sugar, fat, and cholesterol), vitamin family (vitamin A, beta‐cryptoxanthin, zeaxanthin, vitamin B1, vitamin B2, vitamin B6, niacin, total folate, folic acid, vitamin B12, vitamin E, and vitamin C), and microelements (zinc, calcium, phosphorus, sodium, potassium, and selenium) with cancer cachexia (Table S5).

**TABLE 2:** Result from weighted multiple logistic regression between vitamin K intake, other dietary factors, and the risk of cancer cachexia.

Variables	Primary model		Model 1		Model 2		Model 3	
	OR (95% CI)	*p*	OR (95% CI)	*p*	OR (95% CI)	*p*	OR (95% CI)	*p*
Vitamin K intake (quartile)								
Q1	Ref		Ref		Ref		Ref	
Q2	1.59 (0.80, 3.17)	0.18	1.60 (0.79, 3.22)	0.19	1.78 (0.91, 3.50)	0.09	1.79 (0.91, 3.51)	0.09
Q3	1.84 (1.03, 3.29)	0.04	1.85 (1.03, 3.31)	0.04	2.20 (1.26, 3.84)	0.01	2.24 (1.28, 3.92)	0.01
Q4	1.88 (1.05, 3.37)	0.03	1.88 (1.04, 3.41)	0.04	2.11 (1.24, 3.60)	0.01	2.14 (1.25, 3.67)	0.01
*p* for trend		0.04		0.03		0.02		0.02
Energy intake (quartile)								
Q1	Ref		Ref		Ref		Ref	
Q2	0.77 (0.41, 1.47)	0.43	0.77 (0.41, 1.47)	0.43	0.86 (0.44, 1.67)	0.65	0.81 (0.41, 1.57)	0.52
Q3	0.44 (0.21, 0.90)	0.03	0.44 (0.21, 0.90)	0.03	0.55 (0.26, 1.16)	0.12	0.52 (0.25, 1.12)	0.10
Q4	0.77 (0.41, 1.45)	0.42	0.77 (0.41, 1.46)	0.42	1.28 (0.62, 2.62)	0.50	1.16 (0.55, 2.44)	0.70
*p* for trend		0.27		0.27		0.84		1
Fiber intake (quartile)								
Q1	Ref		Ref		Ref		Ref	
Q2	1.55 (0.82, 2.92)	0.18	1.55 (0.81, 2.95)	0.18	1.63 (0.86, 3.06)	0.13	1.83 (0.96, 3.49)	0.07
Q3	2.06 (1.22, 3.50)	0.01	2.07 (1.21, 3.52)	0.01	2.37 (1.38, 4.09)	0.002	2.54 (1.44, 4.47)	0.001
Q4	1.35 (0.71, 2.55)	0.36	1.35 (0.70, 2.59)	0.36	1.72 (0.91, 3.23)	0.09	1.70 (0.87, 3.33)	0.12
*p* for trend		0.25		0.26		0.04		0.03
Alpha‐carotene intake (quartile)								
Q1	Ref		Ref		Ref		Ref	
Q2	0.52 (0.29, 0.92)	0.03	0.52 (0.29, 0.92)	0.02	0.54 (0.29, 0.98)	0.04	0.55 (0.29, 1.02)	0.06
Q3	0.60 (0.31, 1.18)	0.14	0.60 (0.31, 1.16)	0.12	0.62 (0.32, 1.20)	0.15	0.63 (0.32, 1.22)	0.17
Q4	0.87 (0.46, 1.64)	0.67	0.86 (0.47, 1.60)	0.64	0.89 (0.46, 1.69)	0.71	0.86 (0.46, 1.62)	0.64
*p* for trend		0.76		0.74		0.79		0.73
Beta‐carotene intake (quartile)								
Q1	Ref		Ref		Ref		Ref	
Q2	2.13 (1.18, 3.85)	0.01	2.13 (1.17, 3.88)	0.01	2.47 (1.34, 4.53)	0.004	2.62 (1.41, 4.86)	0.002
Q3	1.17 (0.60, 2.30)	0.64	1.17 (0.60, 2.28)	0.65	1.33 (0.67, 2.65)	0.41	1.34 (0.67, 2.67)	0.40
Q4	1.52 (0.85, 2.72)	0.16	1.51 (0.85, 2.69)	0.16	1.55 (0.88, 2.73)	0.12	1.51 (0.87, 2.63)	0.14
*p* for trend		0.65		0.64		0.63		0.74
Food folate intake (quartile)								
Q1	Ref		Ref		Ref		Ref	
Q2	1.67 (0.89, 3.10)	0.11	1.66 (0.89, 3.11)	0.11	1.77 (0.98, 3.22)	0.06	1.80 (0.97, 3.36)	0.06
Q3	1.28 (0.68, 2.40)	0.44	1.28 (0.68, 2.40)	0.45	1.54 (0.80, 2.95)	0.20	1.50 (0.78, 2.88)	0.23
Q4	1.40 (0.82, 2.39)	0.22	1.40 (0.82, 2.39)	0.22	1.86 (1.11, 3.10)	0.02	1.72 (1.01, 2.94)	0.05
*p* for trend		0.55		0.55		0.09		0.18
Lycopene intake (quartile)								
Q1	Ref		Ref		Ref		Ref	
Q2	0.47 (0.23, 0.95)	0.04	0.47 (0.23, 0.95)	0.04	0.45 (0.22, 0.92)	0.03	0.46 (0.22, 0.94)	0.03
Q3	0.70 (0.40, 1.21)	0.20	0.70 (0.40, 1.22)	0.21	0.71 (0.39, 1.31)	0.28	0.70 (0.38, 1.30)	0.26
Q4	0.55 (0.28, 1.08)	0.08	0.55 (0.28, 1.08)	0.08	0.56 (0.28, 1.15)	0.11	0.57 (0.28, 1.14)	0.11
*p* for trend		0.15		0.15		0.22		0.21
Retinol intake (quartile)								
Q1	Ref		Ref		Ref		Ref	
Q2	0.54 (0.30, 0.95)	0.03	0.53 (0.30, 0.95)	0.03	0.56 (0.32, 0.98)	0.04	0.59 (0.33, 1.05)	0.07
Q3	0.86 (0.43, 1.71)	0.66	0.85 (0.42, 1.75)	0.66	0.90 (0.43, 1.87)	0.77	0.91 (0.42, 1.98)	0.81
Q4	0.89 (0.50, 1.59)	0.70	0.89 (0.49, 1.63)	0.70	1.11 (0.59, 2.10)	0.74	1.07 (0.55, 2.05)	0.85
*p* for trend		0.97		0.98		0.56		0.66
Magnesium intake (quartile)								
Q1	Ref		Ref		Ref		Ref	
Q2	1.50 (0.78, 2.87)	0.22	1.50 (0.78, 2.87)	0.22	1.55 (0.83, 2.90)	0.17	1.54 (0.81, 2.92)	0.19
Q3	1.07 (0.60, 1.90)	0.83	1.07 (0.60, 1.90)	0.83	1.28 (0.69, 2.38)	0.43	1.27 (0.68, 2.36)	0.46
Q4	1.87 (1.09, 3.23)	0.02	1.87 (1.09, 3.23)	0.02	2.78 (1.62, 4.76)	< 0.001	2.57 (1.46, 4.53)	0.001
*p* for trend		0.11		0.11		0.01		0.02
Iron intake (quartile)								
Q1	Ref		Ref		Ref		Ref	
Q2	0.46 (0.23, 0.92)	0.03	0.46 (0.23, 0.91)	0.03	0.48 (0.23, 0.98)	0.04	0.44 (0.22, 0.91)	0.03
Q3	0.45 (0.25, 0.82)	0.01	0.45 (0.24, 0.82)	0.01	0.56 (0.29, 1.09)	0.09	0.56 (0.29, 1.08)	0.08
Q4	0.76 (0.39, 1.47)	0.41	0.75 (0.38, 1.48)	0.41	1.09 (0.54, 2.19)	0.81	1.06 (0.51, 2.17)	0.88
*p* for trend		0.44		0.45		0.87		0.88
Copper intake (quartile)								
Q1	Ref		Ref		Ref		Ref	
Q2	1.25 (0.64, 2.47)	0.51	1.25 (0.63, 2.48)	0.51	1.34 (0.69, 2.63)	0.39	1.40 (0.72, 2.75)	0.32
Q3	1.51 (0.84, 2.70)	0.17	1.51 (0.83, 2.71)	0.17	1.83 (0.99, 3.40)	0.05	1.79 (0.95, 3.36)	0.07
Q4	1.42 (0.76, 2.67)	0.27	1.42 (0.76, 2.67)	0.27	1.97 (1.07, 3.61)	0.03	1.86 (1.00, 3.47)	0.05
*p* for trend		0.25		0.25		0.02		0.05

*Note:* Primary model: adjusted for none. Model 1: The age of participants was adjusted. Model 2: The age, sex, education level, race, PIR, and marriage of participants were adjusted. Model 3: The age, sex, education level, race, PIR, smoking status, marriage, drinking status, hypertension, and diabetes mellitus of participants was adjusted.

**FIGURE 2:**
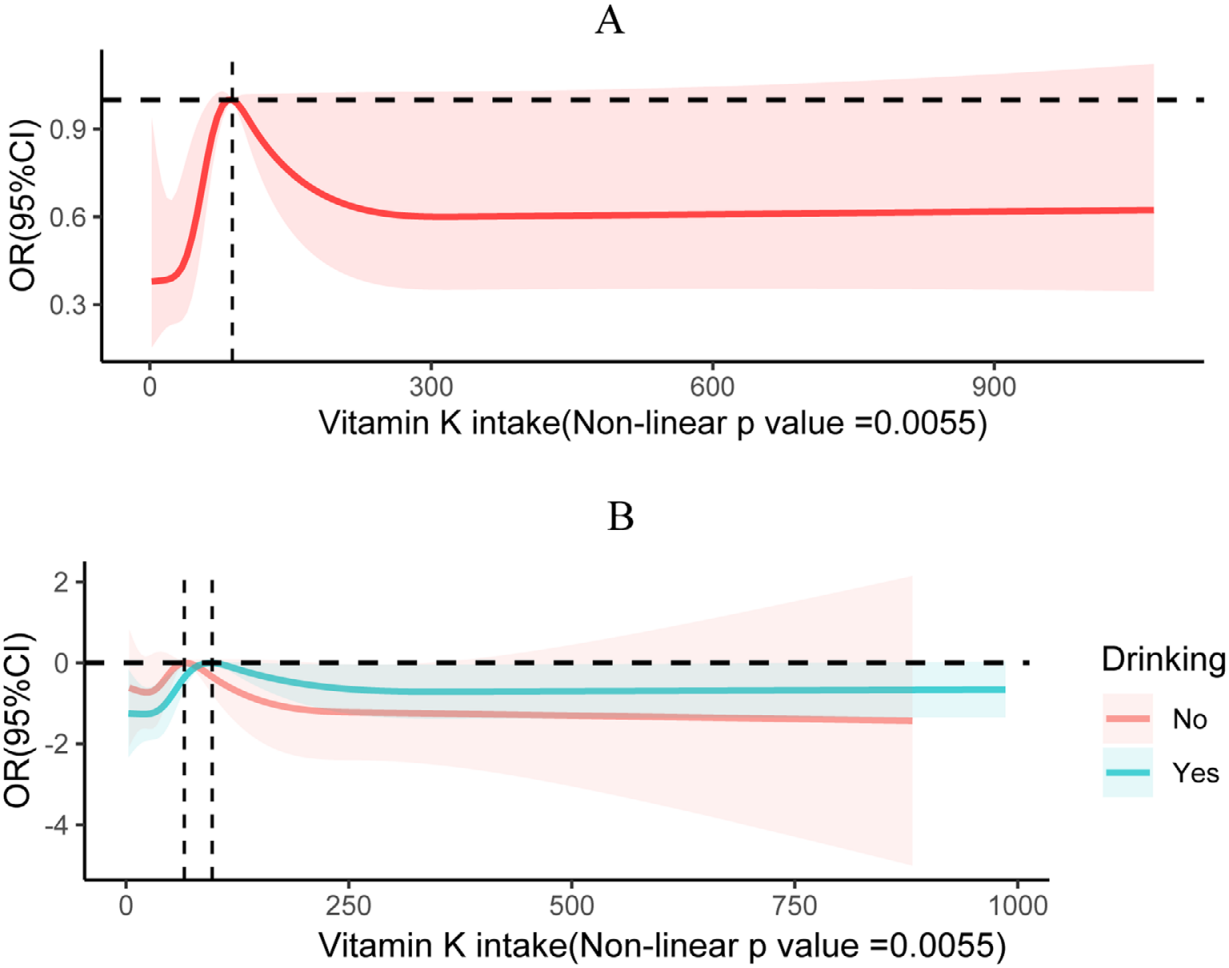
(A) Restricted cubic spline (RCS) curves characterizing the dose–response association between vitamin K intake and cancer cachexia. (B) RCS curves describing the dose–response association between vitamin K intake and cancer cachexia by grouping based on drinking status. The following covariates were adjusted for age, education level, race, PIR, marriage, sex, drinking status, smoking status, hypertension, and diabetes mellitus.

In light of the percentiles of vitamin K intake, all four models demonstrated that the risk of cancer cachexia was higher among those with vitamin K intake in the third and fourth quartiles. Compared to participants in the first quartile of model 3 (odds ratio (OR) = 2.14; 95% confidence interval (95% CI), 1.25–3.67, *p* = 0.01) and (OR = 2.24; 95% CI 1.28–3.92, *p* = 0.01), participants in the fourth and third quartiles had a 114% and 124% elevated risk of cancer cachexia, respectively. Additionally, the incidence of cancer cachexia among participants in the second quartile was higher than that of the lowest quartile, but this association between the variables did not reach statistical significance (OR = 1.79; 95% CI 0.91–3.51, *p* = 0.09). The same conclusion can be drawn from the raw model, model 1, and model 2. Among all models, the most considerable risk of disease was found in the third quartile, where vitamin K intake was between 62.3 to 118.1 mcg/day, indicating the highest risk of cancer cachexia. Furthermore, using the RCS curve to examine the dose–response association between cancer cachexia prevalence and vitamin K intake, we discovered a nonlinear association (nonlinear *p*‐value = 0.0055) between these variables. As vitamin K intake increased, the OR curve for cancer cachexia initially rose sharply, then declined, and finally leveled off, presenting an inverted U‐shaped curve. The study found that the turning point of the OR curve was at a vitamin K intake of 87.875 mcg/day (Figure 2). We also conducted RCS curve analysis on drinking status subgroups and found that the turning point of the OR curve for nondrinking individuals was at a vitamin K intake of 65.475 mcg/day, while for drinking individuals, it was at 96.682 mcg/day, with the analysis conducted under fully adjusted models.

We scrutinized the quartile distribution of dietary energy and fiber intake to elucidate their respective roles in cancer cachexia risk. Individuals within the third quartile of energy intake contained in our findings illustrated a significant reduction in the propensity for cancer cachexia. This negative correlation was consistently observed across the crude model and model 1, with a modest daily caloric consumption ranging from 1778 to 2318 kcals/day correlating with an OR of 0.44, indicating a 56% decrease in cancer cachexia risk (95% CI, 0.21–0.90; *p* = 0.03). In stark contrast, our analysis of dietary fiber intake demonstrated a positive association with the likelihood of cancer cachexia. More specifically, a pronounced correlation was observed between the third quartile of dietary fiber consumption and an increased likelihood of cachexia in every model assessed. Relative to the lowest quartile, this increment in fiber intake (14.1 to 20.5 g/day) was linked to a substantial increase in cancer cachexia risk, ranging from 106% to 154% (OR = 2.54; 95% CI, 1.44–4.47; *p* = 0.001; *p* for trend = 0.03), underscoring the importance of considering fiber intake in the context of cancer cachexia prevention and management.

In our analysis, which examined the percentiles of beta‐carotene, food folate, alpha‐carotene, retinol, and lycopene intake as part of a comprehensive nutritional assessment, we identified a significant increase in the risk of cancer cachexia with the second percentile of beta‐carotene consumption across all models. This increase was substantial, raising the risk by 113%, 113%, 147%, and 162% relative to the lowest percentile, with the highest risk observed in model 3 (95% CI, 1.41–4.86; *p* = 0.002; OR = 2.62), corresponding to a daily intake of 319 to 889 mcg. Additionally, an increased risk was observed with the highest percentile of food folate intake, which was statistically significant in model 2 (OR = 1.86; 95% CI, 1.11–3.10; *p* = 0.02) and model 3 (OR = 1.72; 95% CI, 1.01–2.94; *p* = 0.05). Conversely, alpha‐carotene, retinol, and lycopene intake were found to exert a protective effect against cancer cachexia. Specifically, the second percentile of alpha‐carotene intake was linked with a maximum 46% diminishment in the likelihood of cancer cachexia in comparison with the lowest percentile in the crude model (OR = 0.52; 95% CI, 0.29–0.92; *p* = 0.03), with an intake range of 14 to 57 mcg/day. Similarly, the second percentile of retinol intake was associated with a maximum 47% reduced risk in the crude model (OR = 0.54; 95% CI, 0.30–0.95; *p* = 0.03) and model 1 (OR = 0.53; 95% CI, 0.29–0.92; *p* = 0.03), and a 56% reduction in model 2 (OR = 0.56; 95% CI, 0.32–0.98; *p* = 0.04), corresponding to a daily intake of 187 to 351 mcg. Notably, the second percentile of lycopene intake significantly decreased the risk of cancer cachexia in all models, with the most pronounced effect in model 2 (OR = 0.45; 95% CI, 0.22–0.92; *p* = 0.03), corresponding to an intake range of 1 to 1211 mcg/day.

In the context of iron, copper, and magnesium intake, we observed that the highest percentile of Magnesium consumption significantly elevated the risk of cancer cachexia in all models, with the highest risk observed in model 2 (OR = 2.78; 95% CI, 1.62–4.76; *p* < 0.001, *p* for trend = 0.01), and a similar effect was noted in the model incorporating all adjusted covariates (OR = 2.57; 95% CI, 1.46–4.53; *p* = 0.001, *p* for trend = 0.02), corresponding to a daily intake of 346 to 1480 mg. In contrast, intake of copper at the highest percentile level corresponded to a 97% increased risk for cancer cachexia in model 2 (OR = 0.56; 95% CI, 0.32–0.98; *p* = 0.04, *p* for trend = 0.02), with a corresponding intake range of 1.461–20.095 mg/day. In opposition to these findings, iron intake was associated with a decreased risk of cancer cachexia, with the second percentile of iron intake significantly reducing the risk in all models, with the most notable effect in model 3 (OR = 0.44; 95% CI, 0.22–0.91; *p* = 0.03), corresponding to an intake range of 8.82–12.65 mg/day. Moreover, the third percentile of iron intake in the crude model and model 1 also demonstrated a reduced risk of cancer cachexia.

### 3.3 Diet Vitamin K Intake and Mortality Outcomes

**FIGURE 3:**
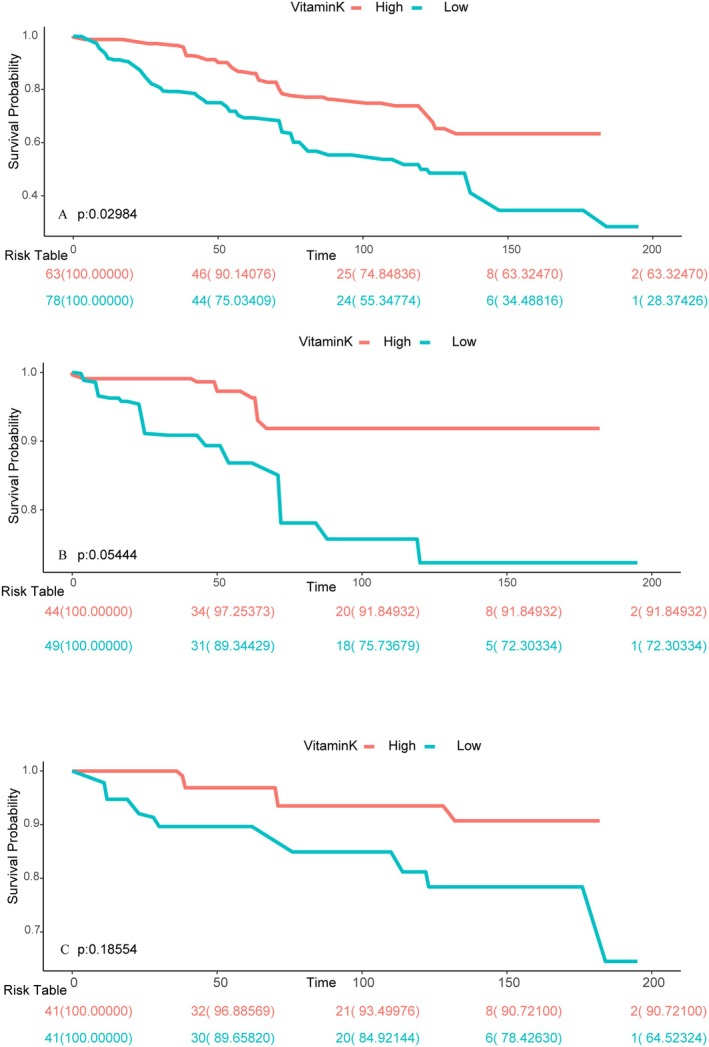
Kaplan–Meier survival curves for mortality outcomes. A for all‐cause mortality, B for cancer mortality, and C for cardiac mortality. According to vitamin K intake among patients with cancer cachexia.

According to the follow‐up results of mortality data, during a mean of 6.2 years of follow‐up, a total of 1188 all‐cause deaths were recorded among the overall cancer population, including 391 fatalities from cancer and 255 cardiac deaths. The cancer cachexia group recorded 94 all‐cause deaths, including 24 cancer deaths and 13 cardiac deaths. The noncancer cachexia group recorded 1116 all‐cause deaths, including 367 cancer deaths and 242 cardiac deaths. The detailed distribution of causes of death and the number of people is shown in Table S3. With adjustments for other factors, vitamin K consumption demonstrated a negative correlation with all‐cause mortality. Results from the Kaplan–Meier survival analysis revealed that the survival rates for all‐cause mortality were significantly different between groups with contrasting vitamin K intake levels (*p* = 0.02984, Figure 3). The multivariate‐adjusted model revealed that compared to individuals with low vitamin K intake, those with high vitamin K intake had significantly lower HR for all‐cause mortality, cancer mortality, and cardiac mortality, with HRs of 0.71 (0.60, 0.84), 0.72 (0.54, 0.97), and 0.64 (0.46, 0.90), respectively (Table 3). Moreover, using the RCS curve to examine the curve association between mortality and vitamin K intake, we found a nonlinear negative correlation between these variables (nonlinear *p*‐value = 0.0371). As vitamin K intake increased, the HR curve for all‐cause mortality dropped sharply and then leveled off, with the study finding a turning point at a vitamin K intake of 185.785 mcg/day (Figure S1), while no curve association was found between cancer mortality and vitamin K (nonlinear *p*‐value = 0.17). Similar conclusions were drawn for noncancer cachexia patients, with the multivariate‐adjusted model showing that compared to individuals with low vitamin K intake, people with high levels of vitamin K ingestion had significantly lower HRs for all‐cause mortality and cardiac mortality, with HRs of 0.73 (0.61, 0.87) and 0.61 (0.43, 0.88), respectively. The risk of cancer mortality was only found to be reduced in model 1 (HR = 0.70; 0.51, 0.95) (Table S4).

**TABLE 3:** Survey‐weighted cox regression analysis was conducted to assess the association between vitamin K intake and mortality among patients with cancer cachexia.

Outcome	Primary model		Model 1		Model 2		Model 3	
	HR (95% CI)	*p*	HR (95% CI)	*p*	HR (95% CI)	*p*	HR (95% CI)	*p*
All‐cause of mortality no. of deaths/patients (94/141)								
Vitamin K intake (quartile)								
Q1	Ref		Ref		Ref		Ref	
Q2	0.90 (0.72, 1.11)	0.30	0.77 (0.64, 0.93)	0.01	0.81 (0.67, 0.97)	0.02	0.82 (0.67, 0.99)	0.04
Q3	0.77 (0.64, 0.94)	0.01	0.66 (0.56, 0.78)	< 0.0001	0.68 (0.57, 0.80)	< 0.0001	0.71 (0.60, 0.84)	< 0.0001
*p* for trend		0.01		< 0.0001		< 0.0001		< 0.0001
Cancer mortality no. of deaths/patients (24/141)								
Vitamin K intake (quartile)								
Q1	Ref		Ref		Ref		Ref	
Q2	0.96 (0.70, 1.32)	0.80	0.80 (0.59, 1.08)	0.14	0.83 (0.61, 1.14)	0.26	0.85 (0.62, 1.16)	0.31
Q3	0.81 (0.59, 1.12)	0.21	0.66 (0.48, 0.89)	0.01	0.69 (0.51, 0.94)	0.02	0.72 (0.54, 0.97)	0.03
*p* for trend		0.2		0.01		0.02		0.03
Cardiac Mortality No. of deaths/patients (94/141)								
Vitamin K intake (quartile)								
Q1	Ref		Ref		Ref		Ref	
Q2	0.83 (0.57, 1.19)	0.30	0.66 (0.47, 0.92)	0.01	0.65 (0.47, 0.89)	0.01	0.64 (0.46, 0.90)	0.01
Q3	0.89 (0.62, 1.28)	0.53	0.68 (0.50, 0.94)	0.02	0.70 (0.51, 0.97)	0.03	0.77 (0.55, 1.09)	0.14
*p* for trend		0.55		0.03		0.04		0.17

*Note:* Primary model: adjusted for none. Model 1: The age of participants was adjusted. Model 2: The age, sex, education level, race, PIR, and marriage of participants were adjusted. Model 3: The age, sex, education level, race, PIR, marriage, drinking status, smoking status, hypertension, and diabetes mellitus of participants was adjusted. Q1, ≤ 38.3 mcg/day; Q2, 38.3–82.3 mcg/day; Q3, ≥ 82.3 mcg/day.

### 3.4 Subgroup Analysis

**FIGURE 4:**
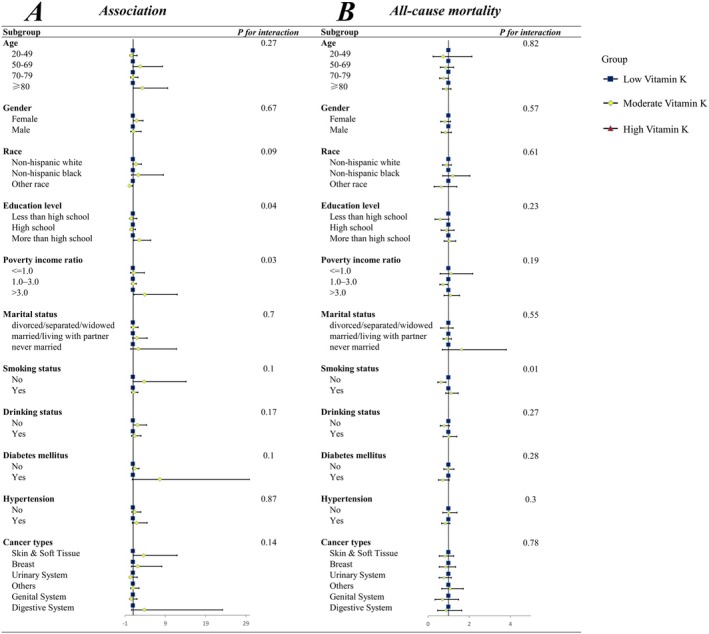
Subgroup analysis forest plot: comparing low and moderate vitamin K intake. Subgroup analyses of the association of vitamin K intake with cancer cachexia (A) and all‐cause mortality (B) among patients with cancer cachexia.

**FIGURE 5:**
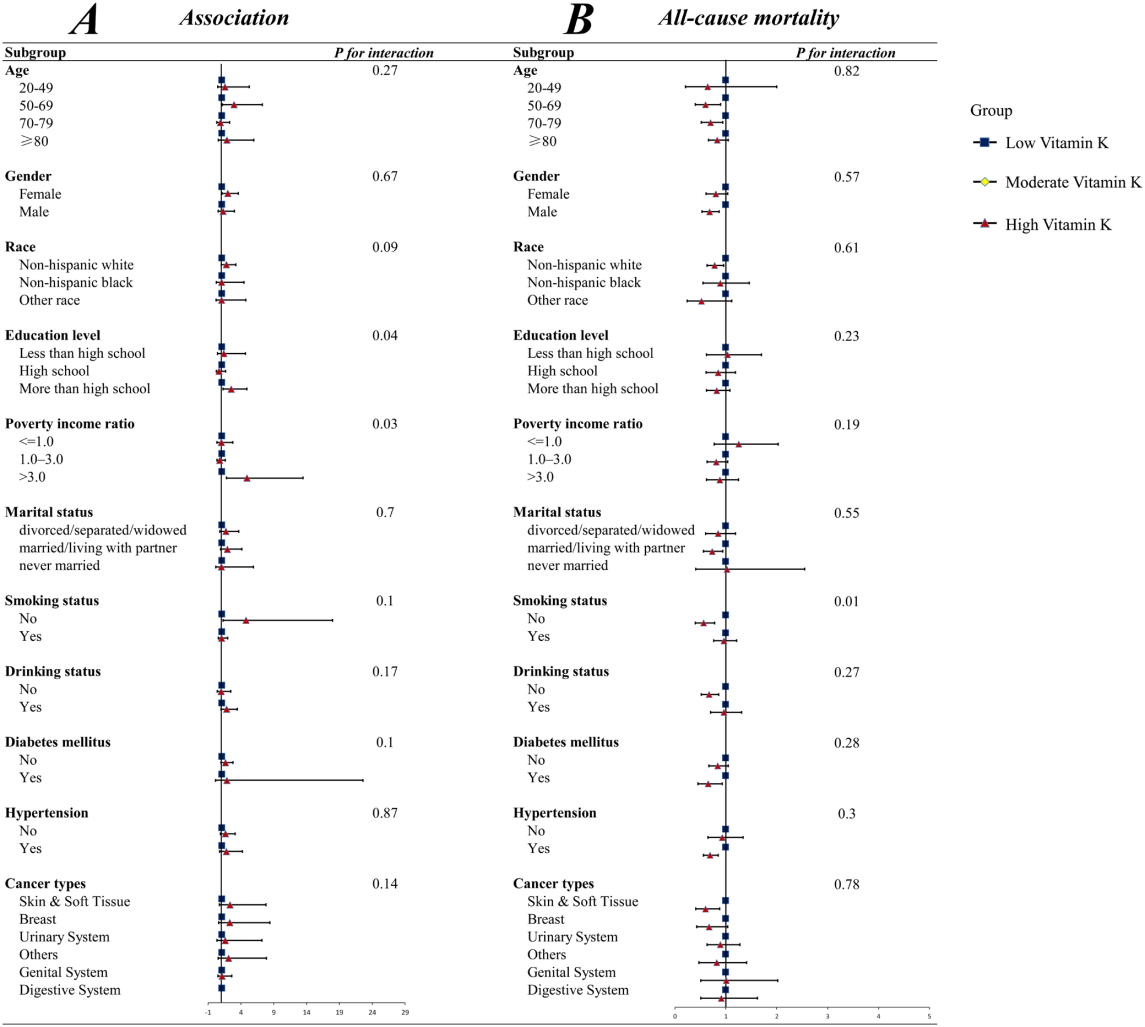
Subgroup analysis forest plot: comparing low and high vitamin K intake. Subgroup analyses of the association of vitamin K intake with cancer cachexia (A) and all‐cause mortality (B) among patients with cancer cachexia.

We used categorized weighted multivariate regression analyses to implement subgroup analyses stratified by age, education level, race, gender, PIR, marital status, drinking status, smoking status, hypertension, diabetes mellitus, and cancer types to further investigate the association between vitamin K intake and cancer cachexia in various populations. Figures 4A and 5A depict subgroup analyses of the association between vitamin K intake and cancer cachexia, and analyze the interaction between vitamin K intake and categorical variables in the study. The results showed a stable positive correlation (*p*‐values > 0.01 for all interactions) between vitamin K intake and the incidence of cancer cachexia in populations with different demographic characteristics, lifestyle habits, and disease conditions. Figures 4B and 5B describe the subgroup analysis of the association between vitamin K intake and all‐cause mortality. There is no significant difference in the association between vitamin K intake and all‐cause mortality among participants of different age, gender, education level, race, PIR, marital status, drinking status, hypertension, diabetes, and cancer types (P for interaction > 0.05). However, we observed a significant interaction between vitamin K intake and smoking status (P for interaction < 0.05).

## 4 Discussion

Within the scope of this research, we included data from 10 cycles of the NHANES database from 1999 to 2018, with a total of 3489 participants, to systematically identify dietary risk factors associated with cancer cachexia and investigate the correlation between dietary intake and mortality in patients with cancer cachexia, especially vitamin K. Cancer cachexia is a comprehensive metabolic chaos that severely affects patients' quality of life and survival (Arends et al. 2021; Sadeghi et al. 2018). By understanding how dietary factors influence cancer cachexia, with particular attention to vitamin K intake, we can provide more targeted nutritional interventions to improve patient prognosis.

In this study, we observed an inverted U‐shaped association between dietary vitamin K intake and the incidence of cancer cachexia. The incidence of cancer cachexia initially increased with increasing vitamin K intake, peaked at approximately 87.9 mcg/day, and then declined with further intake. The underlying mechanism before the turning point may involve vitamin K1‐mediated elevation of Gas6, which exhibits an increase following higher vitamin K1 (Schött et al. 2021). Elevated Gas6 levels promote tumor progression, invasive activity, and angiogenesis, contributing to poorer prognosis (Fusaro et al. 2020; Kirane et al. 2015; Pilli et al. 2020). These pathological processes are strongly linked to the progression of cancer to cancer cachexia (Chen et al. 2025). An additional mechanism involves vitamin K2, which has been shown to enhance glycolysis in cancer cells, resulting in increased lactic acid secretion (Duan et al. 2020). Xiao et al. demonstrated that tumor‐derived lactic acid activates the GPR81 receptor in adipocytes, accelerating lipolysis (Liu et al. 2024). The enhanced lipolysis represents a hallmark feature of cancer cachexia (Molfino et al. 2023). Consequently, vitamin K may increase the incidence of cancer cachexia through the aforementioned mechanisms. Our RCS analysis revealed that, once vitamin K intake surpassed 87.875 mcg/day, the OR declined. Earlier research documented an inverse association between vitamin K intake and INR once intake reached 100 mcg, and circulating coagulation factors II and VII continued to rise with increasing vitamin K intake (Schurgers et al. 2004). Collectively, these findings suggested that vitamin K fostered the synthesis of coagulation factors and attenuated bleeding risk. Because chronic bleeding and its attendant anemia are well‐established drivers of cancer cachexia, the mechanism by which vitamin K mediates bleeding may attenuate the incidence of cancer cachexia (Fearon et al. 2011). The above evidence supports the curve changes of our dose–response association, and the turning point we identified refines this effective range, offering a novel avenue for future clinical research. Malnutrition stands as a significant driver of mortality in cancer cachexia. Its impact arises from the gradual exhaustion of energy stores, the loss of skeletal muscle and visceral proteins, and the resulting failure of vital organs (Kalantar‐Zadeh et al. 2013; Petermann‐Rocha et al. 2022). A recent study suggested that vitamin K can restore intestinal barrier integrity and improve gut health, increasing the absorption of lipids, fat‐soluble vitamins, and essential minerals (Lai et al. 2021). Such improved nutrient assimilation may underlie the reduced mortality of cancer cachexia with increased vitamin K intake.

A cross‐sectional study on dietary nutrient intake and cancer classified cancers into solid and blood cancers (Qin et al. 2025). Through multivariate logistic regression analysis, it demonstrated a positive association between vitamin K intake and cancer, as well as solid cancers among all cancers. Additionally, RCS analysis was used to explore the dose–response association. However, this study did not further investigate the association between dietary nutrient intake and cancer cachexia. Our study, on the other hand, explored the association between dietary nutrient intake and the incidence of cancer cachexia. Meanwhile, we further analyzed the impact of vitamin K intake on the survival prognosis of cancer cachexia survivors. Using RCS analysis, we found an inverse U‐shaped association between vitamin K intake and the development of cancer cachexia, and also explored the potential biological mechanisms before and after the inflection point of the dose–response curve. The results of Kaplan–Meier survival analysis and subgroup analysis further strengthened the stability of the conclusion that vitamin K intake may reduce the mortality rate of cancer cachexia survivors. Although extensive studies have widely explored the importance of nutritional support for patients with cancer cachexia and highlighted the potential benefits of various nutrients such as protein (J. D. Bauer et al. 2006), providing preliminary guidance for clinical practice, there remains a gap in current cross‐sectional studies on dietary intake in relation to human cancer cachexia. Most existing studies are reviews and guideline recommendations concerning dietary nutrition for cancer cachexia (J. D. Bauer et al. 2006; de Campos‐Ferraz et al. 2014; Tanaka et al. 2022), without further investigating the association between dietary nutrient intake and cancer cachexia as well as its prognosis. One study used two mouse models of cancer cachexia to determine the differential effects of a ketogenic diet on tumors and host organisms (Ferrer et al. 2023). This study allowed for precise control of experimental conditions, enabling direct observation of the impact of the ketogenic diet on tumor growth and host physiology. However, mouse models differ biologically from humans and cannot fully simulate human diet and lifestyle, limiting the applicability of the results. In contrast, our study included 3489 US adults, which can reflect real‐world human dietary habits and health status. The large, nationally representative sample enhances statistical power. Meanwhile, research on vitamin K intake and cancer cachexia not only has limited evidence but also lacks clarity on its specific role and recommended dosage in cancer cachexia, thereby restricting its clinical application. In comparison, our study reveals the association between vitamin K intake and the incidence and mortality of cancer cachexia, providing new insights into the dietary management of cancer cachexia.

Our research has several limitations that must be acknowledged. Given the cross‐sectional nature of the NHANES database, causal inference cannot be established. Moreover, specific genetic data of cancer cachexia are unavailable, precluding Mendelian randomization as an alternative approach. The temporal ambiguity makes it difficult to ascertain whether vitamin K intake preceded the onset of cancer cachexia or whether cancer cachexia itself influenced dietary habits. There are certain limitations regarding the definition of cancer cachexia. In this study, only the combination of cancer diagnosis and a participant's BMI < 20 was used as the diagnostic criterion, without incorporating factors such as weight loss and muscle mass. This may introduce some bias into the actual prevalence of cancer cachexia. Although the NHANES dataset is comprehensive in many aspects, it has its own limitations. Nevertheless, our research still provides valuable insights into the nutritional intake risk screening for cancer cachexia and the relationship between vitamin K intake and mortality among cancer cachexia survivors. Moreover, while it is well established that dietary vitamin K intake can be differentiated into subtypes such as vitamin K1 and vitamin K2, that differ in bioavailability and tissue distribution (Bus and Szterk 2021; Halder et al. 2019), and although cancer cachexia is closely related to the characteristics of the tumor, therapeutic exposures, biological mediators, and genetic predispositions, the NHANES database lacks intake data for these specific subtypes and detailed confounding factors. As a result, we could not further investigate the potential association between vitamin K subtypes and the incidence of cancer cachexia, and the aforementioned relevant confounding factors were not included in the analysis. Additionally, the absorption of vitamin K can vary significantly among individuals. Anti‐tumor therapies, including chemotherapy and targeted drugs, may affect bile secretion through mechanisms such as biliary stasis or bile duct inflammation (Grigorian and O'Brien 2014; Mudd and Guddati 2021), thereby potentially reducing the absorption of fat‐soluble vitamins like vitamin K. Consequently, dietary intake data may not always accurately reflect the actual utilization of vitamin K, which could lead to some deviations in our research findings. Furthermore, the rapid progression and low survival rate associated with cancer cachexia may lead to low participation rates or dietary data reported by others, which can cause recall bias or classification errors. In light of these limitations, future research should consider adopting longitudinal cohort studies, prospective trials, and mechanistic investigations to better elucidate the potential causal role of vitamin K in cancer cachexia and to further explore the association between different vitamin K subtypes and cancer cachexia.

## 5 Conclusion

This study reveals a significant inverted U‐shaped association between vitamin K intake and the incidence of cancer cachexia. Specifically, the risk rises with increasing vitamin K intake until it peaks at a threshold, after which it declines. Moreover, among patients with cancer cachexia, higher vitamin K intake is associated with lower all‐cause mortality. Taking into account the combined association between vitamin K intake and the risk of developing cancer cachexia as well as the mortality rate among cancer cachexia survivors, and in conjunction with the recommended vitamin K intake by the US Food and Nutrition Board (Institute of Medicine Panel on 2001), we recommend a daily vitamin K intake exceeding 118.1 mcg/day.

## Author Contributions

In alignment with the CRediT taxonomy framework, the contributions of each author are detailed below: Guoming Chen and Siran Lai led the investigation, data curation, formal analysis, and drafting of the original manuscript. Geer Lin and Tianyi Li each contributed to formal analysis and the drafting of the original manuscript. Cheng Zhang supported the investigation and data curation. Xiangjun Qi, Guang Chen, Guoyi Tang, Ning Wang, and Yibin Feng contributed to writing – review and editing; Yibin Feng additionally provided overall conceptualization. All authors have verified the accuracy of this allocation and approved the final version.

## Ethics Statement

The authors have nothing to report.

## Conflicts of Interest

The authors declare no conflicts of interest.

## Supporting information


**Figure S1:** Restricted cubic splines illustrate the nonlinear associations of vitamin K with mortality from all causes (A) as well as with cancer‐specific mortality (B).
**Table S1:** Detailed description of cancer classification.
**Table S2:** Baseline characteristics among cancer cachexia patients according to vitamin K intake.
**Table S3:** Definition and proportion of causes of death.
**Table S4:** The relationship between dietary vitamin K intake and mortality among noncancer cachexia patients.
**Table S5:** Results from a multiple logistic regression analysis of the association between vitamin K intake, other dietary intake and cancer cachexia, weighted.

## Data Availability

All data in this study are available upon request by contact with the corresponding author.
