# Supplementary material for: Association Between Dietary Vitamin K Intake With Cancer Cachexia and Mortality: NHANES 1999–2018
Source: Food Sci Nutr. 2025 Oct 28;13(11):e70917. doi: 10.1002/fsn3.70917 (PMC12566881; doi:10.1002/fsn3.70917)
Supplement: Supplementary file 1 — Figure S1: Restricted cubic splines illustrate the nonlinear associations of vitamin K with mortality from all causes (A) as well as with cancer‐specific mortality (B). Table S1: Detailed description of cancer classification. Table S2: Baseline characteristics among cancer cachexia patients according to vitamin K intake. Table S3: Definition and proportion of causes of death. Table S4: The relationship between dietary vitamin K intake and mortality among noncancer cachexia patients. Table S5: Results from a multiple logistic regression analysis of the association between vitamin K intake, other dietary intake and cancer cachexia, weighted. [file FSN3-13-e70917-s001.docx]

**
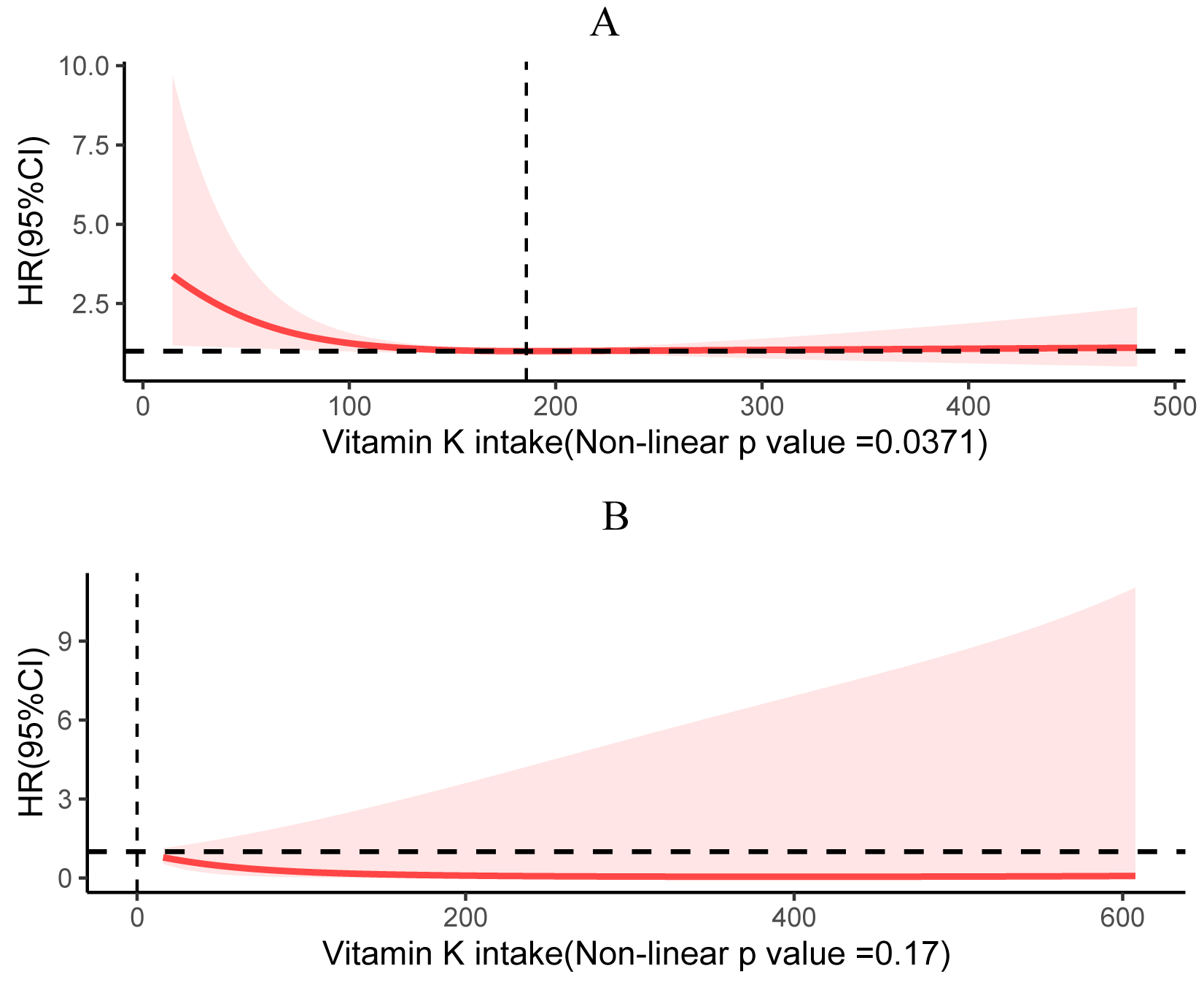
Figure S1** Restricted cubic splines illustrate the non-linear associations of Vitamin K with mortality from all causes (A) as well as with cancer-specific mortality (B)

**Table S1** Detailed description of cancer classification

| Classification | Detailed Location | Count |
| --- | --- | --- |
| Skin & Soft Tissue | Skin (non-melanoma) | 561 |
|  | Melanoma | 202 |
|  | Mouth/tongue/lip | 19 |
|  | Soft tissue (muscle or fat) | 7 |
|  | Skin | 279 |
| Urinary System | Prostate | 527 |
|  | Bladder | 79 |
|  | Kidney | 61 |
| Breast | Breast | 525 |
| Genital System | Cervix (cervical) | 233 |
|  | Uterus (uterine) | 137 |
|  | Ovary (ovarian) | 72 |
|  | Testis (testicular) | 23 |
| Digestive System | Colon | 219 |
|  | Stomach | 18 |
|  | Esophagus (esophageal) | 18 |
|  | Liver | 17 |
|  | Rectum (rectal) | 12 |
|  | Pancreas (pancreatic) | 5 |
|  | Gallbladder | 1 |
| Others | Other | 147 |
|  | Lung | 85 |
|  | Lymphoma/Hodgkin's disease | 79 |
|  | Thyroid | 67 |
|  | Leukemia | 35 |
|  | Bone | 20 |
|  | Larynx/ windpipe | 17 |
|  | Brain | 14 |
|  | Blood | 9 |
|  | Nervous system | 1 |

**Table S2** Baseline characteristics among cancer cachexia patients according to Vitamin K intake

|  | Vitamin K intake | | | | |  |
| --- | --- | --- | --- | --- | --- | --- |
| Variables | Overall | Q1 | Q2 | Q3 | Q4 | P-value |
| N | 141 | 36 | 35 | 35 | 35 |  |
| Age,years | 62.37(1.58) | 60.94(3.73) | 62.79(3.23) | 68.96(2.16) | 56.85(3.40) | 0.05 |
| Sex, n (%) |  |  |  |  |  | 0.99 |
| Female | 96(80.08) | 26(80.83) | 21(77.83) | 25(81.45) | 24(80.33) |  |
| Male | 45(19.92) | 10(19.17) | 14(22.17) | 10(18.55) | 11(19.67) |  |
| Race, n (%) |  |  |  |  |  | 0.44 |
| Non-hispanic white | 107(89.37) | 27(81.02) | 22(89.44) | 33(97.05) | 25(87.89) |  |
| Non-hispanic black | 23( 5.83) | 5(7.40) | 11(9.17) | 2(2.95) | 5(4.40) |  |
| Mexican american | 1( 0.24) | 1(1.24) | 0(0.00) | 0(0.00) | 0(0.00) |  |
| Other race | 10( 4.56) | 3(10.34) | 2( 1.39) | 0( 0.00) | 5( 7.70) |  |
| Education level, n (%) |  |  |  |  |  | < 0.001 |
| Less than high school | 14( 6.14) | 5(15.40) | 3( 4.41) | 2( 2.25) | 4( 4.98) |  |
| High school | 36(16.61) | 15(41.41) | 7( 6.84) | 8(16.15) | 6( 8.87) |  |
| More than high school | 91(77.25) | 16(43.20) | 25(88.75) | 25(81.60) | 25(86.15) |  |
| Poverty income ratio, n (%) |  |  |  |  |  | 0.004 |
| <=1.0 | 27(12.12) | 11(26.32) | 6(12.20) | 4( 6.97) | 6( 7.09) |  |
| 1.0–3.0 | 62(33.32) | 21(58.44) | 18(32.57) | 12(34.61) | 11(15.52) |  |
| >3.0 | 52(54.56) | 4(15.24) | 11(55.24) | 19(58.42) | 18(77.38) |  |
| Marriage, n (%) |  |  |  |  |  | 0.3 |
| Dvorced/separated/widowed | 56(36.54) | 17(50.00) | 13(27.40) | 13(49.37) | 13(23.65) |  |
| Married/living with partner | 72(55.59) | 14(38.60) | 21(63.65) | 19(44.38) | 18(70.39) |  |
| Never married | 13( 7.87) | 5(11.40) | 1( 8.95) | 3( 6.26) | 4( 5.96) |  |
| Energy intake (kcals/day) | 1934.18(109.98) | 1530.40(116.60) | 1954.33(176.93) | 2332.23(264.68) | 1822.70(178.65) | 0.03 |
| Protein intake (g/day) | 73.49(3.96) | 49.89(4.49) | 76.05(9.12) | 82.25(7.58) | 79.23(8.86) | 0.001 |
| Carbohydrate intake (g/day) | 235.26(17.44) | 213.86(18.73) | 231.78(18.91) | 295.86(44.21) | 196.67(26.51) | 0.27 |
| Sugar intake (g/day) | 115.85(13.97) | 113.70(11.82) | 94.47( 7.90) | 169.51(38.70) | 86.75(15.77) | 0.13 |
| Fiber intake (g/day) | 17.17(0.94) | 9.09(1.11) | 17.78(1.37) | 19.51(1.76) | 19.99(2.61) | < 0.0001 |
| Tfat intake (g/day) | 76.98(4.52) | 51.96( 6.13) | 81.21(10.17) | 91.29( 8.95) | 77.03( 7.65) | 0.01 |
| Sfat intake (g/day) | 25.99(2.04) | 18.99(2.64) | 25.86(4.27) | 32.83(5.11) | 24.57(2.35) | 0.09 |
| Mfat intake (g/day) | 27.92(1.68) | 18.57(2.26) | 29.85(3.94) | 32.28(2.85) | 28.52(3.12) | 0.01 |
| Pfat intake (g/day) | 16.31(0.88) | 8.90(1.15) | 18.68(1.60) | 17.93(1.16) | 17.75(2.18) | < 0.0001 |
| Cholesterol intake (mg/day) | 233.90(18.48) | 227.17(41.10) | 201.99(29.68) | 262.44(39.48) | 240.94(32.27) | 0.7 |
| Vitamin A intake (mcg/day) | 708.38(51.88) | 486.12(107.78) | 564.46( 63.47) | 869.01( 98.51) | 842.49(129.90) | 0.03 |
| Retinol intake (mcg/day) | 462.54(38.68) | 418.53(109.72) | 430.03( 61.62) | 593.05( 87.88) | 400.75( 47.53) | 0.22 |
| Alpha-carotene intake (mcg/day) | 451.09(105.94) | 127.69( 55.33) | 206.78( 70.66) | 605.57(215.24) | 751.77(271.99) | 0.05 |
| Beta-carotene intake (mcg/day) | 2683.04(463.65) | 710.09( 255.16) | 1481.69( 395.55) | 2933.11( 638.08) | 4899.66(1253.70) | 0.004 |
| Bcryptoxanthin intake (mcg/day) | 88.50(16.31) | 78.81(36.49) | 77.58(29.89) | 141.32(45.59) | 55.90(13.59) | 0.32 |
| Lycopene intake (mcg/day) | 3816.89(771.21) | 2455.53( 793.50) | 3502.30(1354.66) | 5607.29(1915.77) | 3371.11(1083.15) | 0.36 |
| Zeaxanthin intake (mcg/day) | 1831.52(395.93) | 481.61( 114.70) | 642.38( 80.08) | 1145.64( 118.02) | 4480.62(1210.11) | < 0.001 |
| Vitamin B1 intake (mg/day) | 1.48(0.09) | 1.34(0.24) | 1.67(0.18) | 1.54(0.11) | 1.35(0.17) | 0.58 |
| Vitamin B2 intake (mg/day) | 2.18(0.11) | 1.80(0.27) | 2.24(0.25) | 2.37(0.18) | 2.20(0.23) | 0.43 |
| Niacin intake (mg/day) | 21.88(1.28) | 18.41(2.70) | 24.58(3.22) | 23.02(1.73) | 20.77(2.20) | 0.43 |
| Vitamin B6 intake (mg/day) | 1.76(0.10) | 1.31(0.25) | 1.89(0.22) | 1.95(0.19) | 1.80(0.22) | 0.24 |
| Total folate intake (mcg/day) | 388.92(21.41) | 337.32(65.54) | 416.75(37.94) | 410.15(31.55) | 379.32(40.80) | 0.72 |
| Folic acid intake (mcg/day) | 177.24(21.04) | 209.14(61.46) | 220.28(43.34) | 189.16(35.37) | 105.07(20.97) | 0.02 |
| Food folate intake (mcg/day) | 211.42(11.21) | 128.46(18.51) | 196.53(17.94) | 220.79(17.99) | 273.31(28.90) | < 0.001 |
| Vitamin B12 intake (mg/day) | 4.60(0.31) | 3.91(0.72) | 4.64(0.90) | 5.21(0.65) | 4.45(0.36) | 0.6 |
| Vitamin C intake (mg/day) | 86.42(8.55) | 36.40(11.23) | 92.79(22.49) | 112.49(19.48) | 90.75( 9.76) | < 0.001 |
| Vitamin E intake (mg/day) | 8.98(0.68) | 3.63(0.51) | 8.27(0.81) | 11.21(1.77) | 11.23(1.50) | < 0.0001 |
| Calcium intake (mg/day) | 941.14(79.53) | 546.05( 74.40) | 872.22(147.26) | 1318.93(199.46) | 923.56(104.57) | 0.001 |
| Phosphorus intake (mg/day) | 1274.27(67.79) | 835.82( 79.95) | 1263.89(137.85) | 1512.87(149.98) | 1362.98(158.12) | < 0.001 |
| Magnesium intake (mg/day) | 307.26(14.82) | 187.02(19.76) | 307.67(20.01) | 332.45(24.00) | 366.11(44.22) | < 0.001 |
| Iron intake (mg/day) | 14.33(0.93) | 12.25(1.91) | 16.23(2.15) | 14.12(1.44) | 14.23(1.75) | 0.59 |
| Zinc intake (mg/day) | 11.07(0.67) | 8.56(1.40) | 11.80(1.66) | 12.53(1.20) | 10.77(1.33) | 0.24 |
| Copper intake (mg/day) | 1.31(0.08) | 0.77(0.08) | 1.24(0.10) | 1.40(0.11) | 1.66(0.25) | < 0.001 |
| Sodium intake (mg/day) | 2882.78(156.48) | 2088.12(235.79) | 3309.66(303.80) | 3100.78(288.88) | 2838.06(272.93) | 0.02 |
| Potassium intake (mg/day) | 2601.34(117.34) | 1697.70(166.01) | 2732.76(210.61) | 2902.26(223.67) | 2823.00(269.75) | < 0.001 |
| Selenium intake (mg/day) | 101.27(7.76) | 74.01( 6.61) | 94.62( 8.31) | 114.97(12.43) | 113.32(22.66) | 0.03 |
| Drinking status, n (%) |  |  |  |  |  | 0.04 |
| No | 45(24.78) | 12(29.52) | 18(44.23) | 6(11.61) | 9(16.09) |  |
| Yes | 96(75.22) | 24(70.48) | 17(55.77) | 29(88.39) | 26(83.91) |  |
| Diabetes mellitus, n (%) |  |  |  |  |  | 0.28 |
| No | 129(96.06) | 35(98.07) | 29(91.53) | 32(97.23) | 33(97.70) |  |
| Yes | 12( 3.94) | 1(1.93) | 6(8.47) | 3(2.77) | 2(2.30) |  |
| Hypertension, n (%) |  |  |  |  |  | 0.61 |
| No | 76(62.17) | 22(66.39) | 16(64.18) | 16(50.66) | 22(68.15) |  |
| Yes | 65(37.83) | 14(33.61) | 19(35.82) | 19(49.34) | 13(31.85) |  |
| Smoking status, n (%) |  |  |  |  |  | 0.18 |
| No | 40(40.50) | 5(17.52) | 9(38.89) | 12(41.08) | 14(57.24) |  |
| Yes | 101(59.50) | 31(82.48) | 26(61.11) | 23(58.92) | 21(42.76) |  |
| Vitamin K intake (mcg/day) | 130.09(22.32) | 24.02( 2.13) | 56.47( 1.44) | 96.36( 3.49) | 301.42(66.80) | < 0.0001 |

Continuous variables are shown as weighted means±standard errors.

Categorical variables are shown as unweighted counts (weighted percentages).

Q1,≤34.5 mcg/day; Q2,34.5–62.3 mcg/day; Q3,62.3–118.1 mcg/day; Q4, ≥ 118.1 mcg/day.

**Table S3** Definition and proportion of causes of death

| Total cancer patients (n = 3489) | | |
| --- | --- | --- |
| Cause of death (ICD-10 code) | Count | Percent (%) |
| Malignant neoplasms (C00-C97) | 391 | 32.9 |
| All other causes (residual) | 273 | 23.0 |
| Diseases of heart (I00-I09, I11, I13, I20-I51) | 255 | 21.5 |
| Chronic lower respiratory diseases (J40-J47) | 71 | 6.0 |
| Cerebrovascular diseases (I60-I69) | 58 | 4.9 |
| Alzheimer's disease (G30) | 43 | 3.6 |
| Accidents (unintentional injuries) (V01-X59, Y85-Y86) | 32 | 2.7 |
| Influenza and pneumonia (J09-J18) | 24 | 2.0 |
| Diabetes mellitus (E10-E14) | 22 | 1.9 |
| Nephritis, nephrotic syndrome and nephrosis (N00-N07, N17-N19, N25-N27) | 19 | 1.5 |
| Total | 1188 | 100 |
| Cancer cachexia patients (n = 141) | | |
| Cause of death (ICD-10 code) | Count | Percent (%) |
| Malignant neoplasms (C00-C97) | 24 | 25.5 |
| Diabetes mellitus (E10-E14) | 22 | 23.4 |
| All other causes (residual) | 16 | 17.0 |
| Diseases of heart (I00-I09, I11, I13, I20-I51) | 13 | 13.8 |
| Chronic lower respiratory diseases (J40-J47) | 9 | 9.6 |
| Alzheimer's disease (G30) | 4 | 4.3 |
| Accidents (unintentional injuries) (V01-X59, Y85-Y86) | 3 | 3.2 |
| Cerebrovascular diseases (I60-I69) | 1 | 1.1 |
| Influenza and pneumonia (J09-J18) | 1 | 1.1 |
| Nephritis, nephrotic syndrome and nephrosis (N00-N07, N17-N19, N25-N27) | 1 | 1.1 |
| Total | 94 | 100 |
| Non cancer cachexia patients (n = 3348) | | |
| Cause of death (ICD-10 code) | Count | Percent (%) |
| Malignant neoplasms (C00-C97) | 367 | 32.9 |
| All other causes (residual) | 257 | 23.0 |
| Diseases of heart (I00-I09, I11, I13, I20-I51) | 242 | 21.7 |
| Chronic lower respiratory diseases (J40-J47) | 62 | 5.6 |
| Cerebrovascular diseases (I60-I69) | 57 | 5.1 |
| Alzheimer's disease (G30) | 39 | 3.5 |
| Accidents (unintentional injuries) (V01-X59, Y85-Y86) | 29 | 2.6 |
| Influenza and pneumonia (J09-J18) | 23 | 2.1 |
| Diabetes mellitus (E10-E14) | 22 | 2.0 |
| Nephritis, nephrotic syndrome and nephrosis (N00-N07, N17-N19, N25-N27) | 18 | 1.6 |
| Total | 1116 | 100 |

**Table S4** The relationship between dietary vitamin K intake and mortality among non-cancer cachexia patients

| Outcome | Primary model | | Model 1 | | Model 2 | | Model 3 | |
| --- | --- | --- | --- | --- | --- | --- | --- | --- |
|  | HR (95% CI) | P value | HR (95% CI) | P value | HR (95% CI) | P value | HR (95% CI) | P value |
| All-Cause of Mortality No. of deaths/patients (94/141) | | | | | | | | |
| Vitamin K intake (quartile) | | | | | | | | |
| Q1 | ref |  | ref |  | ref |  | ref |  |
| Q2 | 0.88(0.72,1.09) | 0.25 | 0.77(0.64,0.93) | 0.01 | 0.80(0.66,0.96) | 0.02 | 0.81(0.66,0.98) | 0.03 |
| Q3 | 0.81(0.67,0.99) | 0.04 | 0.69(0.58,0.82) | <0.0001 | 0.70(0.58,0.83) | <0.0001 | 0.73(0.61,0.87) | <0.001 |
| p for trend |  | 0.04 |  | <0.0001 |  | <0.0001 |  | <0.001 |
| Cancer Mortality No. of deaths/patients (24/141) | | | | | | | | |
| Vitamin K intake (quartile) | | | | | | | | |
| Q1 | ref |  | ref |  | ref |  | ref |  |
| Q2 | 0.90(0.65,1.24) | 0.52 | 0.75(0.55,1.03) | 0.08 | 0.79(0.57,1.08) | 0.14 | 0.80(0.58,1.11) | 0.18 |
| Q3 | 0.87(0.63,1.19) | 0.38 | 0.70(0.51,0.95) | 0.02 | 0.73(0.54,1.00) | 0.05 | 0.76(0.56,1.02) | 0.06 |
| p for trend |  | 0.38 |  | 0.03 |  | 0.05 |  | 0.07 |
| Cardiac Mortality No. of deaths/patients (94/141) | | | | | | | | |
| Vitamin K intake (quartile) | | | | | | | | |
| Q1 | ref |  | ref |  | ref |  | ref |  |
| Q2 | 0.78(0.53,1.15) | 0.21 | 0.63(0.44,0.89) | 0.01 | 0.62(0.44,0.87) | 0.01 | 0.61(0.43,0.88) | 0.01 |
| Q3 | 0.94(0.64,1.38) | 0.75 | 0.71(0.51,1.00) | 0.05 | 0.73(0.52,1.02) | 0.07 | 0.80(0.55,1.15) | 0.23 |
| p for trend |  | 0.77 |  | 0.08 |  | 0.1 |  | 0.27 |

Primary model: adjusted for none.

Model 1: The age of participants were adjusted.

Model 2:The age, sex, education level, race, PIR and marriage of participants were adjusted.

Model 3:The age, sex, education level, race, PIR, marriage, drinking status, smoking status, hypertension and diabetes mellitus of participants were adjusted.

Q1,≤38.3 mcg/day; Q2,38.3–82.3 mcg/day; Q3,≥ 82.3 mcg/day.

**Table S5** Results from a multiple logistic regression analysis of the association between vitamin K intake, other dietary intake and cancer cachexia, weighted.

| Variables | Primary model | | Model 1 | | Model 2 | | Model 3 | |
| --- | --- | --- | --- | --- | --- | --- | --- | --- |
|  | OR (95% CI) | P value | OR (95% CI) | P value | OR (95% CI) | P value | OR (95% CI) | P value |
| Protein intake (quartile) | | | | | | | | |
| Q1 | ref |  | ref |  | ref |  | ref |  |
| Q2 | 0.87(0.44,1.71) | 0.68 | 0.87(0.44,1.71) | 0.68 | 0.91(0.46,1.81) | 0.78 | 0.88(0.44,1.78) | 0.73 |
| Q3 | 0.65(0.31,1.37) | 0.25 | 0.65(0.31,1.37) | 0.25 | 0.85(0.40,1.84) | 0.69 | 0.86(0.39,1.91) | 0.71 |
| Q4 | 0.80(0.44,1.45) | 0.47 | 0.81(0.45,1.46) | 0.47 | 1.27(0.66,2.45) | 0.46 | 1.21(0.61,2.40) | 0.57 |
| p for trend |  | 0.37 |  | 0.37 |  | 0.59 |  | 0.66 |
| Carbohydrate intake(quartile) | | | | | | | | |
| Q1 | ref |  | ref |  | ref |  | ref |  |
| Q2 | 0.77(0.37,1.61) | 0.49 | 0.77(0.37,1.60) | 0.48 | 0.81(0.38,1.74) | 0.59 | 0.77(0.36,1.62) | 0.48 |
| Q3 | 0.74(0.40,1.38) | 0.34 | 0.74(0.40,1.37) | 0.34 | 0.86(0.47,1.58) | 0.62 | 0.82(0.43,1.56) | 0.54 |
| Q4 | 0.97(0.49,1.92) | 0.93 | 0.97(0.49,1.94) | 0.94 | 1.36(0.66,2.80) | 0.40 | 1.24(0.60,2.58) | 0.56 |
| p for trend |  | 0.93 |  | 0.93 |  | 0.43 |  | 0.57 |
| Sugar intake (quartile) | | | | | | | | |
| Q1 | ref |  | ref |  | ref |  | ref |  |
| Q2 | 0.83(0.41,1.68) | 0.61 | 0.83(0.41,1.66) | 0.59 | 0.84(0.42,1.67) | 0.62 | 0.77(0.39,1.50) | 0.44 |
| Q3 | 0.90(0.45,1.79) | 0.76 | 0.90(0.44,1.81) | 0.76 | 0.96(0.47,1.96) | 0.91 | 0.81(0.39,1.68) | 0.57 |
| Q4 | 0.88(0.45,1.71) | 0.70 | 0.88(0.45,1.72) | 0.70 | 1.09(0.55,2.16) | 0.81 | 0.98(0.49,1.96) | 0.95 |
| p for trend |  | 0.75 |  | 0.75 |  | 0.77 |  | 0.95 |
| Tfat intake(quartile) | | | | | | | | |
| Q1 | ref |  | ref |  | ref |  | ref |  |
| Q2 | 1.52(0.82,2.84) | 0.18 | 1.52(0.82,2.84) | 0.18 | 1.69(0.90,3.16) | 0.10 | 1.71(0.91,3.22) | 0.09 |
| Q3 | 0.95(0.53,1.69) | 0.85 | 0.95(0.53,1.69) | 0.85 | 1.12(0.62,2.03) | 0.71 | 1.12(0.61,2.05) | 0.72 |
| Q4 | 1.12(0.61,2.05) | 0.71 | 1.12(0.62,2.04) | 0.70 | 1.72(0.91,3.23) | 0.09 | 1.63(0.86,3.12) | 0.14 |
| p for trend |  | 0.8 |  | 0.81 |  | 0.3 |  | 0.39 |
| Sfat intake(quartile) | | | | | | | | |
| Q1 | ref |  | ref |  | ref |  | ref |  |
| Q2 | 0.88(0.47,1.65) | 0.69 | 0.88(0.46,1.66) | 0.68 | 0.96(0.51,1.83) | 0.90 | 0.94(0.49,1.80) | 0.86 |
| Q3 | 0.69(0.35,1.37) | 0.29 | 0.69(0.35,1.38) | 0.29 | 0.81(0.40,1.64) | 0.56 | 0.80(0.40,1.62) | 0.54 |
| Q4 | 0.98(0.51,1.88) | 0.96 | 0.98(0.51,1.88) | 0.96 | 1.42(0.70,2.88) | 0.33 | 1.37(0.66,2.84) | 0.39 |
| p for trend |  | 0.86 |  | 0.86 |  | 0.44 |  | 0.51 |
| Mfat intake(quartile) | | | | | | | | |
| Q1 | ref |  | ref |  | ref |  | ref |  |
| Q2 | 1.09(0.56,2.12) | 0.79 | 1.09(0.56,2.11) | 0.79 | 1.21(0.61,2.40) | 0.59 | 1.18(0.60,2.32) | 0.62 |
| Q3 | 0.90(0.54,1.51) | 0.70 | 0.90(0.54,1.52) | 0.70 | 1.07(0.61,1.87) | 0.81 | 0.99(0.56,1.77) | 0.98 |
| Q4 | 1.15(0.62,2.15) | 0.66 | 1.15(0.61,2.16) | 0.66 | 1.79(0.91,3.52) | 0.09 | 1.66(0.83,3.32) | 0.15 |
| p for trend |  | 0.8 |  | 0.8 |  | 0.16 |  | 0.25 |
| Pfat intake(quartile) | | | | | | | | |
| Q1 | ref |  | ref |  | ref |  | ref |  |
| Q2 | 1.02(0.53,1.94) | 0.96 | 1.02(0.53,1.95) | 0.96 | 1.06(0.55,2.04) | 0.87 | 1.06(0.54,2.07) | 0.87 |
| Q3 | 1.16(0.65,2.06) | 0.61 | 1.16(0.65,2.06) | 0.61 | 1.33(0.74,2.38) | 0.34 | 1.32(0.73,2.40) | 0.35 |
| Q4 | 0.90(0.46,1.75) | 0.75 | 0.90(0.46,1.75) | 0.75 | 1.19(0.60,2.35) | 0.62 | 1.11(0.55,2.22) | 0.77 |
| p for trend |  | 0.86 |  | 0.86 |  | 0.46 |  | 0.6 |
| Cholesterol intake(quartile) | | | | | | | | |
| Q1 | ref |  | ref |  | ref |  | ref |  |
| Q2 | 0.82(0.43,1.55) | 0.54 | 0.82(0.43,1.56) | 0.54 | 0.87(0.45,1.67) | 0.67 | 0.89(0.46,1.70) | 0.72 |
| Q3 | 0.77(0.40,1.47) | 0.42 | 0.77(0.40,1.47) | 0.42 | 0.94(0.49,1.82) | 0.86 | 0.99(0.50,1.95) | 0.97 |
| Q4 | 0.74(0.37,1.46) | 0.38 | 0.74(0.37,1.46) | 0.38 | 0.98(0.48,2.00) | 0.95 | 0.93(0.46,1.87) | 0.84 |
| p for trend |  | 0.38 |  | 0.38 |  | 0.99 |  | 0.91 |
| Vitamin A intake(quartile) | | | | | | | | |
| Q1 | ref |  | ref |  | ref |  | ref |  |
| Q2 | 1.10(0.57,2.12) | 0.77 | 1.11(0.58,2.11) | 0.76 | 1.17(0.61,2.24) | 0.63 | 1.23(0.64,2.37) | 0.52 |
| Q3 | 1.41(0.70,2.83) | 0.33 | 1.42(0.69,2.93) | 0.34 | 1.58(0.74,3.36) | 0.23 | 1.57(0.71,3.46) | 0.26 |
| Q4 | 1.52(0.86,2.68) | 0.15 | 1.53(0.85,2.75) | 0.16 | 1.75(0.96,3.21) | 0.07 | 1.67(0.88,3.15) | 0.11 |
| p for trend |  | 0.12 |  | 0.14 |  | 0.06 |  | 0.11 |
| Bcryptoxanthin intake(quartile) | | | | | | | | |
| Q1 | ref |  | ref |  | ref |  | ref |  |
| Q2 | 0.59(0.30,1.16) | 0.12 | 0.59(0.30,1.15) | 0.12 | 0.61(0.31,1.20) | 0.15 | 0.58(0.29,1.15) | 0.12 |
| Q3 | 0.72(0.40,1.30) | 0.27 | 0.71(0.40,1.26) | 0.24 | 0.73(0.41,1.30) | 0.28 | 0.69(0.38,1.26) | 0.23 |
| Q4 | 0.60(0.31,1.17) | 0.13 | 0.59(0.31,1.14) | 0.12 | 0.61(0.31,1.18) | 0.14 | 0.55(0.28,1.10) | 0.09 |
| p for trend |  | 0.18 |  | 0.15 |  | 0.17 |  | 0.11 |
| Zeaxanthin intake(quartile) | | | | | | | | |
| Q1 | ref |  | ref |  | ref |  | ref |  |
| Q2 | 1.12(0.57,2.23) | 0.74 | 1.12(0.56,2.23) | 0.74 | 1.19(0.59,2.38) | 0.62 | 1.06(0.54,2.07) | 0.87 |
| Q3 | 0.79(0.43,1.46) | 0.45 | 0.79(0.42,1.47) | 0.45 | 0.84(0.45,1.60) | 0.60 | 0.81(0.42,1.55) | 0.52 |
| Q4 | 1.35(0.79,2.31) | 0.27 | 1.35(0.78,2.32) | 0.28 | 1.40(0.82,2.40) | 0.21 | 1.30(0.76,2.22) | 0.34 |
| p for trend |  | 0.49 |  | 0.49 |  | 0.41 |  | 0.51 |
| Zeaxanthin intake(quartile) | | | | | | | | |
| Q1 | ref |  | ref |  | ref |  | ref |  |
| Q2 | 1.12(0.57,2.23) | 0.74 | 1.12(0.56,2.23) | 0.74 | 1.19(0.59,2.38) | 0.62 | 1.06(0.54,2.07) | 0.87 |
| Q3 | 0.79(0.43,1.46) | 0.45 | 0.79(0.42,1.47) | 0.45 | 0.84(0.45,1.60) | 0.60 | 0.81(0.42,1.55) | 0.52 |
| Q4 | 1.35(0.79,2.31) | 0.27 | 1.35(0.78,2.32) | 0.28 | 1.40(0.82,2.40) | 0.21 | 1.30(0.76,2.22) | 0.34 |
| p for trend |  | 0.49 |  | 0.49 |  | 0.41 |  | 0.51 |
| Vitamin B1 intake(quartile) | | | | | | | | |
| Q1 | ref |  | ref |  | ref |  | ref |  |
| Q2 | 0.77(0.38,1.56) | 0.46 | 0.77(0.38,1.56) | 0.46 | 0.85(0.41,1.77) | 0.66 | 0.81(0.39,1.68) | 0.57 |
| Q3 | 0.93(0.50,1.74) | 0.83 | 0.93(0.50,1.74) | 0.82 | 1.16(0.60,2.26) | 0.65 | 1.18(0.60,2.34) | 0.62 |
| Q4 | 0.79(0.38,1.65) | 0.53 | 0.79(0.37,1.66) | 0.53 | 1.23(0.56,2.70) | 0.61 | 1.22(0.54,2.76) | 0.63 |
| p for trend |  | 0.65 |  | 0.65 |  | 0.49 |  | 0.48 |
| Vitamin B2 intake(quartile) | | | | | | | | |
| Q1 | ref |  | ref |  | ref |  | ref |  |
| Q2 | 0.73(0.37,1.47) | 0.38 | 0.73(0.36,1.47) | 0.38 | 0.80(0.40,1.62) | 0.54 | 0.75(0.37,1.55) | 0.44 |
| Q3 | 0.91(0.44,1.89) | 0.81 | 0.91(0.44,1.90) | 0.81 | 1.13(0.53,2.40) | 0.76 | 1.01(0.46,2.20) | 0.98 |
| Q4 | 1.03(0.59,1.79) | 0.93 | 1.03(0.58,1.80) | 0.93 | 1.60(0.87,2.93) | 0.13 | 1.40(0.74,2.65) | 0.30 |
| p for trend |  | 0.72 |  | 0.73 |  | 0.1 |  | 0.23 |
| Niacin intake(quartile) | | | | | | | | |
| Q1 | ref |  | ref |  | ref |  | ref |  |
| Q2 | 0.78(0.37,1.65) | 0.51 | 0.78(0.37,1.65) | 0.51 | 0.85(0.39,1.84) | 0.67 | 0.82(0.38,1.78) | 0.62 |
| Q3 | 0.84(0.40,1.74) | 0.64 | 0.84(0.40,1.74) | 0.64 | 1.07(0.50,2.30) | 0.85 | 1.02(0.47,2.20) | 0.96 |
| Q4 | 0.72(0.39,1.35) | 0.30 | 0.72(0.39,1.34) | 0.30 | 1.11(0.56,2.17) | 0.77 | 1.05(0.53,2.08) | 0.88 |
| p for trend |  | 0.37 |  | 0.37 |  | 0.65 |  | 0.76 |
| Vitamin B6 intake(quartile) | | | | | | | | |
| Q1 | ref |  | ref |  | ref |  | ref |  |
| Q2 | 0.88(0.42,1.86) | 0.74 | 0.88(0.42,1.86) | 0.74 | 0.93(0.44,1.97) | 0.85 | 0.99(0.46,2.15) | 0.99 |
| Q3 | 0.89(0.53,1.52) | 0.68 | 0.89(0.52,1.53) | 0.68 | 1.10(0.62,1.96) | 0.73 | 1.15(0.62,2.12) | 0.66 |
| Q4 | 0.65(0.35,1.20) | 0.17 | 0.65(0.35,1.20) | 0.17 | 0.93(0.49,1.76) | 0.83 | 0.93(0.48,1.78) | 0.82 |
| p for trend |  | 0.19 |  | 0.19 |  | 0.99 |  | 0.96 |
| Total folate intake(quartile) | | | | | | | | |
| Q1 | ref |  | ref |  | ref |  | ref |  |
| Q2 | 1.19(0.61,2.35) | 0.60 | 1.19(0.61,2.35) | 0.61 | 1.32(0.66,2.64) | 0.43 | 1.24(0.60,2.57) | 0.56 |
| Q3 | 1.15(0.57,2.32) | 0.70 | 1.15(0.57,2.33) | 0.70 | 1.38(0.66,2.89) | 0.39 | 1.30(0.60,2.82) | 0.50 |
| Q4 | 1.14(0.56,2.32) | 0.72 | 1.14(0.55,2.34) | 0.73 | 1.58(0.74,3.34) | 0.23 | 1.49(0.68,3.26) | 0.31 |
| p for trend |  | 0.77 |  | 0.78 |  | 0.23 |  | 0.31 |
| Folic acid intake(quartile) | | | | | | | | |
| Q1 | ref |  | ref |  | ref |  | ref |  |
| Q2 | 0.52(0.26,1.06) | 0.07 | 0.52(0.26,1.06) | 0.07 | 0.57(0.28,1.16) | 0.12 | 0.56(0.27,1.14) | 0.11 |
| Q3 | 0.94(0.53,1.66) | 0.82 | 0.94(0.53,1.66) | 0.82 | 1.06(0.59,1.90) | 0.85 | 1.07(0.59,1.93) | 0.83 |
| Q4 | 0.90(0.49,1.64) | 0.72 | 0.89(0.49,1.65) | 0.72 | 1.15(0.62,2.12) | 0.66 | 1.16(0.61,2.20) | 0.65 |
| p for trend |  | 0.92 |  | 0.92 |  | 0.43 |  | 0.41 |
| Vitamin B12 intake(quartile) | | | | | | | | |
| Q1 | ref |  | ref |  | ref |  | ref |  |
| Q2 | 0.71(0.35,1.46) | 0.35 | 0.71(0.35,1.46) | 0.35 | 0.77(0.37,1.58) | 0.47 | 0.76(0.36,1.59) | 0.46 |
| Q3 | 1.19(0.65,2.17) | 0.58 | 1.18(0.64,2.20) | 0.59 | 1.37(0.73,2.57) | 0.32 | 1.37(0.72,2.62) | 0.34 |
| Q4 | 0.75(0.44,1.29) | 0.30 | 0.75(0.43,1.30) | 0.30 | 0.98(0.56,1.70) | 0.94 | 1.03(0.58,1.82) | 0.93 |
| p for trend |  | 0.75 |  | 0.75 |  | 0.52 |  | 0.44 |
| Vitamin C intake(quartile) | | | | | | | | |
| Q1 | ref |  | ref |  | ref |  | ref |  |
| Q2 | 0.65(0.30,1.42) | 0.28 | 0.65(0.30,1.42) | 0.27 | 0.66(0.31,1.44) | 0.30 | 0.69(0.33,1.44) | 0.32 |
| Q3 | 0.92(0.45,1.91) | 0.83 | 0.92(0.44,1.93) | 0.83 | 0.96(0.45,2.03) | 0.91 | 0.99(0.46,2.12) | 0.98 |
| Q4 | 1.19(0.63,2.25) | 0.59 | 1.19(0.64,2.21) | 0.58 | 1.28(0.70,2.37) | 0.42 | 1.27(0.72,2.25) | 0.41 |
| p for trend |  | 0.42 |  | 0.41 |  | 0.29 |  | 0.28 |
| Vitamin E intake(quartile) | | | | | | | | |
| Q1 | ref |  | ref |  | ref |  | ref |  |
| Q2 | 0.77(0.42,1.42) | 0.40 | 0.77(0.42,1.41) | 0.40 | 0.84(0.44,1.61) | 0.59 | 0.78(0.39,1.56) | 0.48 |
| Q3 | 0.94(0.50,1.75) | 0.84 | 0.94(0.50,1.75) | 0.84 | 1.11(0.58,2.12) | 0.75 | 1.01(0.51,1.97) | 0.99 |
| Q4 | 1.38(0.78,2.45) | 0.27 | 1.38(0.77,2.46) | 0.27 | 1.81(0.98,3.35) | 0.06 | 1.70(0.88,3.27) | 0.11 |
| p for trend |  | 0.19 |  | 0.2 |  | 0.04 |  | 0.07 |
| Calcium intake(quartile) | | | | | | | | |
| Q1 | ref |  | ref |  | ref |  | ref |  |
| Q2 | 0.60(0.33,1.09) | 0.09 | 0.60(0.33,1.08) | 0.09 | 0.60(0.32,1.11) | 0.10 | 0.59(0.31,1.13) | 0.11 |
| Q3 | 0.74(0.39,1.41) | 0.36 | 0.74(0.39,1.41) | 0.36 | 0.76(0.39,1.50) | 0.43 | 0.75(0.38,1.50) | 0.42 |
| Q4 | 0.83(0.45,1.54) | 0.56 | 0.83(0.45,1.54) | 0.56 | 1.00(0.51,1.96) | 1.00 | 1.02(0.50,2.10) | 0.95 |
| p for trend |  | 0.74 |  | 0.74 |  | 0.85 |  | 0.82 |
| Phosphorus intake(quartile) | | | | | | | | |
| Q1 | ref |  | ref |  | ref |  | ref |  |
| Q2 | 0.70(0.36,1.38) | 0.31 | 0.70(0.36,1.38) | 0.31 | 0.74(0.37,1.46) | 0.38 | 0.72(0.36,1.44) | 0.35 |
| Q3 | 0.59(0.32,1.09) | 0.09 | 0.59(0.32,1.08) | 0.09 | 0.72(0.38,1.40) | 0.33 | 0.72(0.37,1.42) | 0.34 |
| Q4 | 0.85(0.44,1.64) | 0.63 | 0.85(0.44,1.64) | 0.63 | 1.26(0.63,2.53) | 0.51 | 1.20(0.57,2.53) | 0.64 |
| p for trend |  | 0.61 |  | 0.61 |  | 0.57 |  | 0.65 |
| Zinc intake(quartile) | | | | | | | | |
| Q1 | ref |  | ref |  | ref |  | ref |  |
| Q2 | 0.64(0.33,1.22) | 0.17 | 0.64(0.33,1.22) | 0.17 | 0.67(0.34,1.32) | 0.25 | 0.66(0.34,1.30) | 0.23 |
| Q3 | 0.58(0.30,1.11) | 0.10 | 0.58(0.30,1.11) | 0.10 | 0.71(0.35,1.44) | 0.34 | 0.74(0.36,1.49) | 0.39 |
| Q4 | 0.93(0.49,1.77) | 0.83 | 0.93(0.49,1.77) | 0.83 | 1.44(0.72,2.86) | 0.30 | 1.48(0.73,3.01) | 0.28 |
| p for trend |  | 0.85 |  | 0.85 |  | 0.37 |  | 0.33 |
| Sodium intake(quartile) | | | | | | | | |
| Q1 | ref |  | ref |  | ref |  | ref |  |
| Q2 | 0.84(0.46,1.55) | 0.58 | 0.84(0.46,1.55) | 0.58 | 0.92(0.50,1.69) | 0.79 | 1.02(0.55,1.89) | 0.96 |
| Q3 | 0.60(0.30,1.19) | 0.14 | 0.60(0.30,1.19) | 0.14 | 0.72(0.36,1.45) | 0.36 | 0.73(0.36,1.48) | 0.38 |
| Q4 | 0.59(0.30,1.15) | 0.12 | 0.59(0.30,1.15) | 0.12 | 0.91(0.45,1.83) | 0.79 | 0.92(0.46,1.86) | 0.82 |
| p for trend |  | 0.08 |  | 0.08 |  | 0.6 |  | 0.59 |
| Potassium intake(quartile) | | | | | | | | |
| Q1 | ref |  | ref |  | ref |  | ref |  |
| Q2 | 1.50(0.75,3.00) | 0.25 | 1.49(0.74,3.01) | 0.26 | 1.60(0.79,3.23) | 0.19 | 1.64(0.81,3.33) | 0.17 |
| Q3 | 0.82(0.43,1.56) | 0.54 | 0.82(0.43,1.55) | 0.54 | 0.97(0.49,1.93) | 0.94 | 0.91(0.45,1.85) | 0.79 |
| Q4 | 0.93(0.53,1.64) | 0.81 | 0.93(0.53,1.63) | 0.80 | 1.37(0.75,2.51) | 0.31 | 1.29(0.68,2.43) | 0.43 |
| p for trend |  | 0.26 |  | 0.25 |  | 0.69 |  | 0.95 |
| Selenium intake(quartile) | | | | | | | | |
| Q1 | ref |  | ref |  | ref |  | ref |  |
| Q2 | 0.86(0.46,1.63) | 0.65 | 0.86(0.46,1.63) | 0.65 | 0.92(0.49,1.75) | 0.81 | 0.91(0.48,1.74) | 0.79 |
| Q3 | 0.78(0.38,1.60) | 0.50 | 0.78(0.38,1.60) | 0.50 | 0.96(0.46,1.99) | 0.92 | 0.98(0.47,2.04) | 0.95 |
| Q4 | 0.76(0.37,1.52) | 0.43 | 0.76(0.38,1.52) | 0.43 | 1.16(0.56,2.40) | 0.69 | 1.16(0.55,2.41) | 0.70 |
| p for trend |  | 0.42 |  | 0.42 |  | 0.74 |  | 0.72 |

Primary model: adjusted for none.

Model 1: The age of participants were adjusted.

Model 2:The age, sex, education level, race, PIR and marriage of participants were adjusted.

Model 3:The age, sex, education level, race, PIR, marriage, drinking status, smoking status, hypertension and diabetes mellitus of participants were adjusted.
